# *miR-509-5p* and *miR-1243* increase the sensitivity to gemcitabine by inhibiting epithelial-mesenchymal transition in pancreatic cancer

**DOI:** 10.1038/s41598-017-04191-w

**Published:** 2017-06-21

**Authors:** Hidekazu Hiramoto, Tomoki Muramatsu, Daisuke Ichikawa, Kousuke Tanimoto, Satoru Yasukawa, Eigo Otsuji, Johji Inazawa

**Affiliations:** 1 Department of Molecular Cytogenetics, Medical Research Institute, Tokyo Medical and Dental University (TMDU), Tokyo, Japan; 2 Bioresource Research Center, TMDU, Tokyo, Japan; 3 Department of Digestive Surgery, Graduate School of Medical Science, Kyoto Prefectural University of Medicine, Kyoto, Japan; 4 Genome Laboratory, Medical Research Institute, TMDU, Tokyo, Japan; 5 Department of Pathology, Kyoto Prefectural University of Medicine, Kyoto, Japan; 6 First Department of Surgery, Faculty of Medicine, University of Yamanashi, Yamanashi, Japan

## Abstract

The epithelial-mesenchymal transition (EMT) contributes to various processes in cancer progression, such as metastasis and drug resistance. Since we have already established a cell-based reporter system for identifying EMT-suppressive microRNAs (miRNAs) in the pancreatic cancer cell line Panc1, we performed a function-based screening assay by combining this reporter system and a miRNA library composed of 1,090 miRNAs. As a result, we identified *miR-509-5p* and *miR-1243* as EMT-suppressive miRNAs, although the mechanisms for EMT-suppression induced by these miRNAs have yet to be clarified. Herein, we demonstrated that overexpression of *miR-509-5p* and *miR-1243* increased the expression of E-cadherin through the suppression of EMT-related gene expression and that drug sensitivity increased with a combination of each of these miRNAs and gemcitabine. Moreover, *miR-509-5p* was associated with worse overall survival in patients with pancreatic cancer and was identified as an independently selected predictor of mortality. Our findings suggest that *miR-509-5p* and *miR-1243* might be novel chemotherapeutic targets and serve as biomarkers in pancreatic cancer.

## Introduction

Pancreatic cancer is the most lethal and common cancer in the world^1^. According to the National Cancer Research Center Japan, the mortality rate from pancreatic cancer is the fourth highest among all cancers in Japan in 2014 (http://ganjoho.jp/reg_stat/statistics/stat/summary.html). Pancreatic ductal adenocarcinoma (PDAC) accounts for 90% of pancreatic cancers, and its prognosis remains very poor, with a 5-year survival of 7–8% to date. Most PDACs are diagnosed at an already advanced stage because there is currently no reliable diagnostic method for the diagnosis of early stage PDAC. Advanced-stage PDAC is characterized by invasion of the lymph nodes and vasculature or distant metastasis^2^. The current therapeutic strategies of chemotherapy and/or radiation therapy provide limited effects for such advanced PDAC^3^. In the last decade, microRNAs (miRNAs) have become firmly established as critical molecules in normal and neoplastic cells^4^. Thus, a detailed understanding of the miRNA-based molecular mechanisms by which pancreatic cancer is so malignant might provide useful insights into the identification of biomarkers and development of novel therapeutic strategies for this virulent tumor.

Epithelial-mesenchymal transition (EMT) plays important roles in cell differentiation, wound healing, and fibrosis in normal tissue and during embryonic development^5^. EMT also contributes to cancer progression, including invasion, metastasis and chemo-resistance^6^. When an epithelial cell is stimulated by EMT inducers, it can transform into a mesenchymal cell with the ability to migrate and invade into other tissues and organs^7^. This phenotypic transition is triggered by several extracellular signals, such as TGF-β and WNT, resulting in an activation of EMT-promoting transcription factors, such as the ZEB family, the Snail family, and Twist^8^. In addition, cancer cells can acquire stem cell-like characteristics and chemoresistant properties through EMT^9^. However, because EMT has phenotypic plasticity, a mesenchymal cell can transform into an epithelial cell through a mesenchymal-to-epithelial transition (MET)^10^. Taken together, inhibiting EMT is a potential therapeutic strategy for cancers.

miRNAs are endogenous, small non-coding, single-stranded RNAs of 20–25 nucleotides that regulate the expression of target genes at the post-transcriptional level by binding to complementary sequences within the 3′ untranslated region (3′ UTR) of their target gene mRNAs^4^. An individual miRNA usually has multiple target genes with partially complementary mRNA sequences, whereas a single gene can be targeted by several miRNAs^11^. miRNAs play crucial roles in the modulation of various biological processes, including EMT^12, 13^. In addition, miRNAs act as tumor suppressors and/or oncogenes in a cell type-dependent manner in various cancers^14^. A number of miRNAs function as crucial modulators of cell proliferation, migration and EMT^15–17^. For example, the *miR-200* family inhibits EMT through the direct suppression of ZEB1/2 and increases the sensitivity of cancer cells to chemotherapeutic agents^18, 19^. Using a cell-based reporter assay system with the *CDH1* promoter, we identified *miR-655* as both an EMT-suppressive miRNA and a predictor for poor prognosis in esophageal squamous cell carcinoma^20^. EMT-related miRNAs might be important biomarkers for diagnosis of several cancer including pancreatic cancer and contribute to overcoming chemoresistance via combination therapy using anti-cancer drugs^11, 21^.

In the present study, we identified two novel EMT-suppressive miRNAs, *miR-509-5p* and *miR-1243*, through a combination of our cell-based reporter system and a miRNA library containing 1,090 synthetic miRNAs. We also clarified their functions, including the direct targets of each miRNA. Our screening system has already proved useful for the identification of miRNAs related to the phenotypic transformation of EMT^20, 22^. We demonstrated that *miR-509-5p* induced an MET phenotype by directly regulating *VIM* and *HMGA2*. By contrast, *miR-1243* directly regulated *SMAD2* and *SMAD4*, which regulate the TGF-β signaling pathway, resulting in an induction of the MET phenotype. In addition, we found that those miRNAs could increase the sensitivity of the pancreatic cancer cell line Panc1 to gemcitabine. Interestingly, the expression of *miR-509-5p* was significantly associated with a worse overall survival in patients with pancreatic cancer and was indicated as an independently selected predictor for overall survival. Taken together, our findings suggest that a novel therapeutic strategy for pancreatic cancer might involve a combination of gemcitabine and *miR-509-5p* or *miR-1243*, which regulate EMT, and that *miR-509-5p* might be useful as a prognostic biomarker in pancreatic cancer.

## Results

### EMT-suppressive miRNAs were extracted using a cell-based reporter system and miRNA library in Panc1 cells

**Figure 1:**
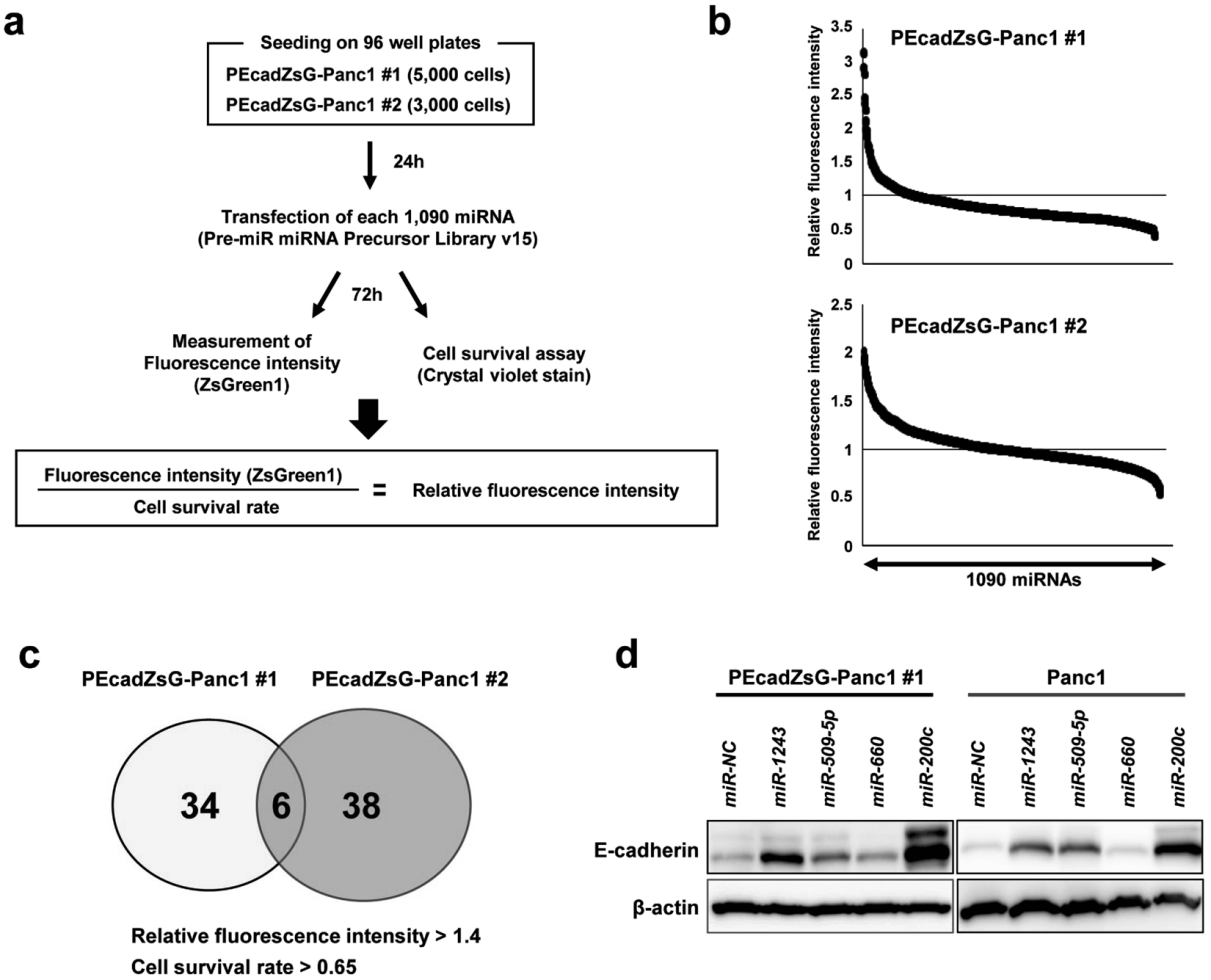
EMT-suppressive miRNAs are extracted using the function-based screening by combining the cell-based reporter system and an miRNA library in Panc1 clones. (**a**) Flow chart of the study. (**b**) Result of the function-based screening using Pre-miRTM miRNA Precursor Library-Human V15, which contains 1,090 miRNAs mimicking human mature miRNAs. The fluorescence intensity of ZsGreen1 was measured in duplicate with a fluorescence microplate reader. The relative fluorescence intensity in each transfectant was calculated using the fluorescence intensity in negative control cells and was normalized based on the cell survival rate, as measured by the crystal violet stain (Supplementary Table S1). The lower closed arrow indicates the 1090 miRNAs examined. (**c**) Venn diagram showing the overlap of six miRNAs between PEcadZsG-Panc1 #1 and #2. (**d**) The expression of E-cadherin in PEcadZsG-Panc1 #1 and parental Panc1 cells transfected with each candidate miRNA that was selected in the screening.

We previously established a cell-based reporter system with the *CDH1/*E-cadherin promoter in Panc1 cells (Supplementary Fig. S1)^20^. In the present study, we established two clones (PEcadZsG-Panc1 #1 and #2) that expressed ZsGreen1 protein by increased *CDH1* promoter activity in Panc1 cells. To identify EMT-suppressive miRNAs, we performed a function-based screening by combining our cell-based reporter assay system and a miRNA library containing 1,090 miRNAs. A different number of cells were cultured for each clone because each clone had a different cell growth ability. After transfection of each miRNA in this library, we measured the cell growth and fluorescence intensity and calculated the relative fluorescence intensity (RFI) (Fig. 1a). Figure 1b shows the results of this screening in PEcadZsG-Panc1 #1 (upper) and #2 (bottom) 72 hours after transfection with each miRNA. We established the criteria for extracting EMT-suppressive miRNAs (RFI >1.4, cell survival rate >0.65) and then extracted 40 miRNAs from PEcadZsG-Panc1 #1 and 44 miRNAs from PEcadZsG-Panc1 #2 (Fig. 1c and Supplementary Table S1). Six miRNAs, *miR-200c*, miR-367*, miR-452*, *miR-509-5p*, *miR-660* and *miR-1243*, which were common in both screening assays, were identified as candidate EMT-suppressive miRNAs (Table 1). miR-367* and miR-452* were excluded from these candidate miRNAs because these miRNAs are expressed at low levels in the body^23^. We next validated whether the remaining four candidate miRNAs increased the expression of E-cadherin in PEcadZsG-Panc1 #1 and Panc1 cells. Overexpression of *miR-200c*, *miR-509-5p* and *miR-1243* induced the expression of endogenous E-cadherin in both cell lines (Fig. 1d). Because little has been reported about *miR-509-5p* and *miR-1243* with respect to the EMT phenotype, we focused on the novel function of these miRNAs as EMT-suppressive miRNAs.

**Table 1:** Summary of six miRNAs selected as candidates for EMT-suppressive miRNAs in functional-based screening using stable Panc1 clones transfected with a reporter construct containing a promoter sequence of CDH1/E-cadherin in the upstream region of the ZsGreen1 reporter gene and Pre-miRTM miRNA Precursor Library - Human V15 (Ambion).

Pre-miR^TM^ miRNA Precursor	Mature Sequence	Ratio of fluorescence intensity of ZsGreen1 (RFI)﻿^#﻿^		Ratio of growth level (RG)﻿^##﻿^		Relative fluorescence intensity (RFI/RG)	
		Panc1#1	Panc1#2	Panc1#1	Panc1#2	Panc1#1	Panc1#2
*hsa-miR-200c*	UAAUACUGCCGGGUAAUGAUGGA	3.362	1.594	1.072	0.941	3.121	1.825
hsa-miR-367*	ACUGUUGCUAAUAUGCAACUCU	2.176	1.206	0.939	0.754	2.317	1.620
hsa-miR-452*	CUCAUCUGCAAAGAAGUAAGUG	1.153	1.237	0.691	0.740	1.671	1.659
*hsa-miR-509-5p*	UACUGCAGACAGUGGCAAUCA	1.603	1.136	0.980	0.810	1.635	1.405
*hsa-miR-660*	UACCCAUUGCAUAUCGGAGUUG	1.153	1.481	0.804	1.036	1.438	1.439
*hsa-miR-1243*	AACUGGAUCAAUUAUAGGAGUG	1.247	1.118	0.799	0.656	1.600	1.705

^#^RFI in cells 72 hours after transfection with each miRNA was normalized to that in miR-NC transfectants.

^##^RG of viable cells assessed by cristal biolet staining 72 hours after transfection with miRNAs. This assay was employed to normalize the number of viable cells relative to the control transfectants.

### *miR-509-5p* and *miR-1243* induced the MET phenotype through the suppression of ZEB1 and Snail

**Figure 2:**
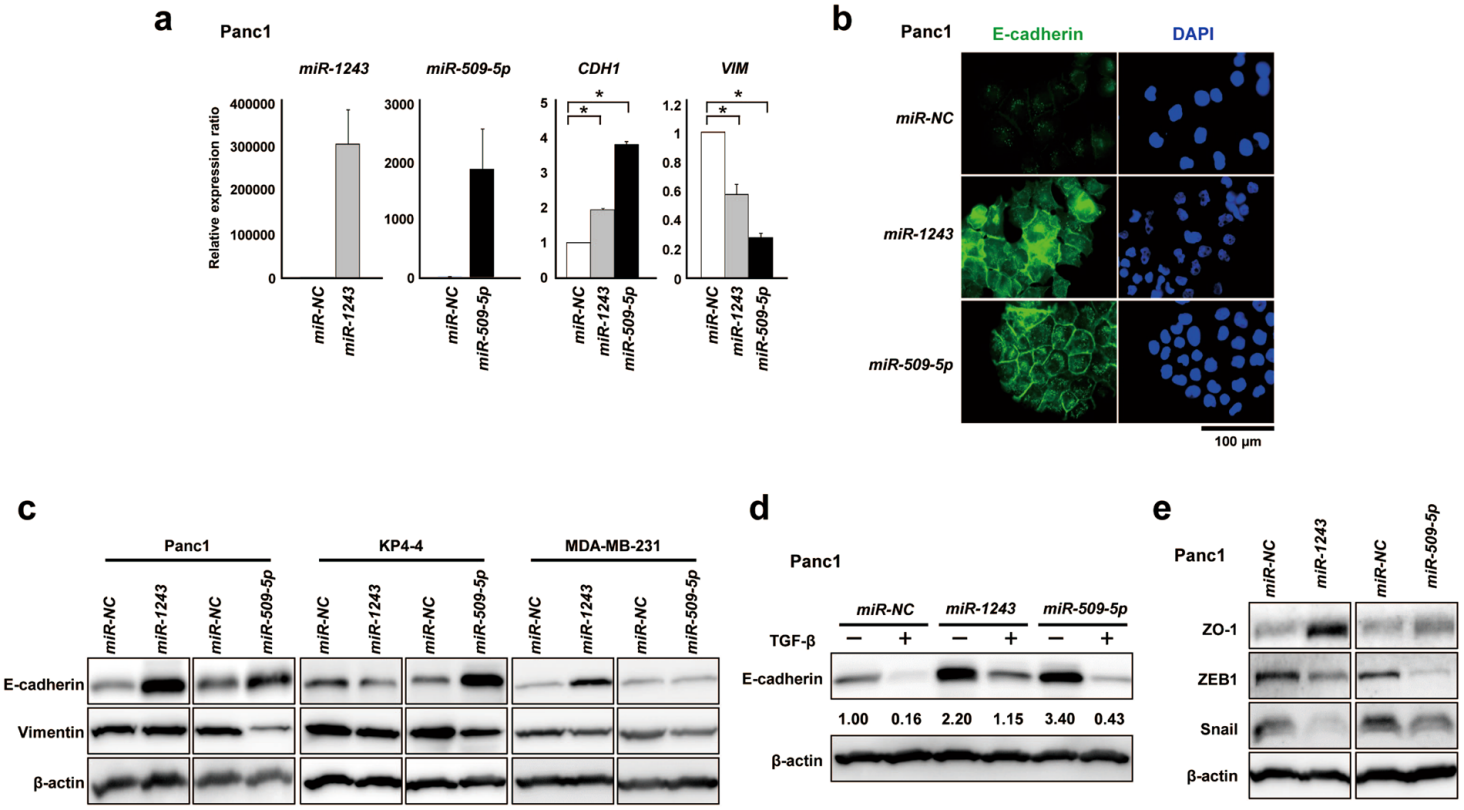
*miR-509-5p* and *miR-1243* induce an MET phenotype in several cancer cell lines. (**a**) The expression of *miR-509-5p*, *miR-1243* and EMT marker genes was confirmed by qRT-PCR in Panc1 cells 72 hours after transfection of *miR-509-5p* and *miR-1243*; the data are the mean ± SD (bars) for duplicate experiments. (**b**) Immunofluorescent staining of E-cadherin (green) and DAPI (blue) in Panc1 cells after transfection of 10 nmol/L of *miR-NC*, *miR-509-5p* or *miR-1243*. (**c**) Western blot analysis of E-cadherin and Vimentin protein levels in Panc1, KP4-4 and MDA-MB-231 cells 72 hours after transfection of 10 nmol/L of *miR-NC*, *miR-509-5p* or *miR-1243*. (**d**) The expression of E-cadherin in Panc1 cells 72 hours after treatment with or without TGF-β (5 ng/ml) and simultaneous transfection with *miR-NC*, *miR-509-5p* and *miR-1243*. The number under the figure indicates the relative ratio based on the *miR-NC* expression level. (**e**) Western blot analysis of the EMT-related proteins ZO-1, ZEB1 and Snail in Panc1 cells 72 hours after transient transfection with 10 nmol/L of *miR-NC*, *miR-509-5p* and *miR-1243*. Student’s *t*-test was used for statistical analysis, and asterisks represent *P* < 0.05 versus *miR-NC* transfectants.

We first evaluated the expression of *miR-509-5p* and *miR-1243* in 24 pancreatic cancer cell lines. The expression levels of these miRNAs tended to be lower in these cells compared with that in normal pancreatic tissue (Supplementary Fig. S2), suggesting that these miRNAs have tumor suppressive functions. To investigate the mechanism by which these miRNAs induced an MET phenotype, we evaluated the EMT marker genes E-cadherin, Vimentin, ZO-1, ZEB1 and Snail in several cancer cell lines, Panc1, KP4-4, SU.86.86, BxPC3 and MDA-MB-231 (Figs 2 and S3). Overexpression of these miRNAs increased the expression of E-cadherin at both the mRNA and protein levels in Panc1 cells, whereas the expression of Vimentin was reduced by these miRNAs (Fig. 2a–c). In addition, MDA-MB-231 cells, a breast cancer cell line, also exhibited an MET phenotype when transfected with *miR-1243*, resulting in the upregulation of E-cadherin and the downregulation of Vimentin. However, overexpression of *miR-509-5p* yielded no increase in the expression of E-cadherin (Fig. 2c). Interestingly, in KP4-4 cells, a *SMAD4*-depleted pancreatic cancer cell line, the MET phenotype was induced by *miR-509-5p* but not by *miR-1243*, suggesting that the mechanism by which the MET phenotype is induced by *miR-1243* might depend on the TGF-β signaling pathway (Fig. 2c). Therefore, to probe whether *miR-1243* depends on the TGF-β signaling pathway to induce the MET phenotype, we analyzed E-cadherin expression in Panc1 cells using TGF-β recombinant protein, which induces an EMT phenotype through the activation of the TGF-β signaling pathway^24^. As a result, we observed that the expression of E-cadherin in both *miR-NC* and *miR-509-5p* transfectants was suppressed by TGF-β, whereas the effect of TGF-β was weak in *miR-1243* transfectants compared with *miR-﻿Negative Control (﻿miR- NC)* transfectants (Fig. 2d). These results indicate that *miR-1243* induced the MET phenotype through the regulation of the TGF-β signaling pathway. We also checked the expression of ZO-1, ZEB1 and Snail, the representative EMT marker genes, in Panc1 cells transfected with each of the miRNAs and observed that the protein expression level of ZO-1 was increased by these miRNAs, whereas that of ZEB1 and Snail was reduced (Fig. 2e).

### *miR-509-5p* and *miR-1243* suppressed cell migration and invasion and targeted EMT-related genes

**Figure 3:**
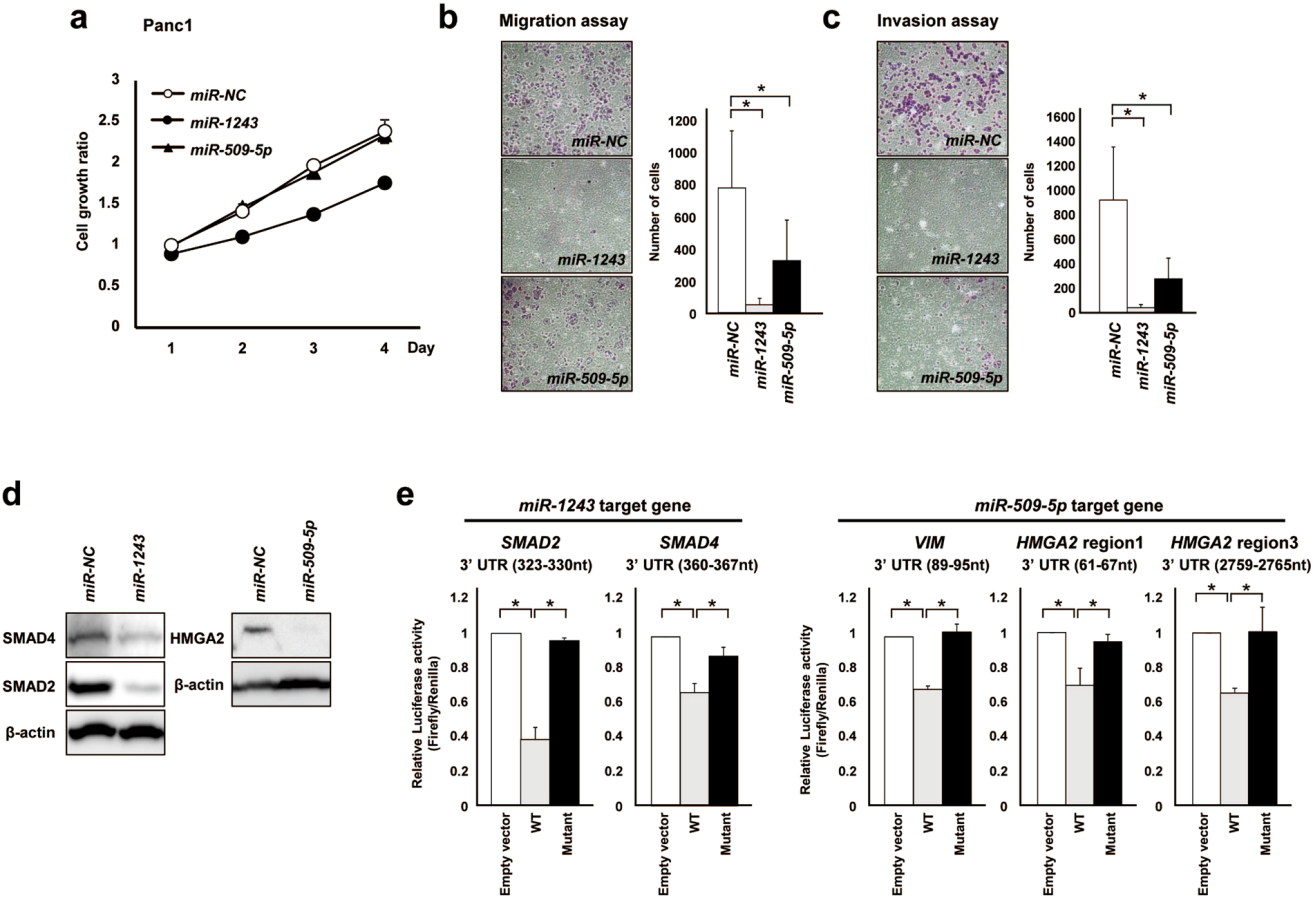
*miR-509-5p* and *miR-1243* suppress cell motility and invasion through an MET phenotype alteration and directly target *VIM*, *HMGA2*, *SMAD2* and *SMAD4*. (**a**) The number of viable cells 24–96 hours after transfection with 5 nmol/L of *miR-NC*, *miR-509-5p* and *miR-1243* in Panc1 cells was assessed by the WST-8 assay. Each data point represents the mean of triplicate experiments (bars, SD). (**b**,**c**) Transwell migration and invasion assays were performed in 24-well modified Boyden chambers without and with Matrigel, respectively. Panc1 cells (4 × 10^4^ cells per well [migration and invasion assay]) that were transfected with each miRNA were transferred into the upper chamber, and the migrated or invaded cells on the lower surface of the filters were fixed, stained and counted after 24 hours of incubation. Experiments were performed in triplicate, and each data point represents the mean (bars, SD). (**d**) Western blot analysis of SMAD2, SMAD4 and HMGA2 in Panc1 cells 72 hours after transfection with 10 nmol/L of *miR-NC*, *miR-509-5p* and *miR-1243*. (**e**) Results of the luciferase reporter assays in Panc1 cells after co-transfection of the pmirGLO Dual-Luciferase vectors containing wild-type (WT) of *SMAD2*, *SMAD4*, *VIM* and *HMGA2* or mutant variants of these genes and *miR-NC*, *miR-509-5p* and *miR-1243*. Student’s *t*-test was used for statistical analysis, and asterisks represent *P* < 0.05 versus *miR-NC* transfectants.

To evaluate the functions of *miR-509-5p* and *miR-1243*, we utilized several biological approaches, including cell growth, migration and invasion assays. Overexpression of *miR-509-5p* did not affect cell growth in Panc1 cells, but *miR-1243* suppressed cell growth (Fig. 3a). These results were consistent with the first screening (Table 1). The cell migration and invasion abilities in *miR-509-5p* or *miR-1243* transfectants were reduced compared with *miR-NC* transfectants (Fig. 3b,c). To explore the mechanism through which these miRNAs could suppress migration and invasion abilities, we sought to determine their direct target genes using the TargetScan database. We extracted *SMAD2*, *SMAD4*, *VIM*, *ZEB1* and *HMGA2* as candidate target genes for each miRNA. *SMAD2* and *SMAD4* contain target sequences of *miR-1243* and are well-known regulators of the TGF-β signaling pathway^25, 26^. *VIM*, *ZEB1* and *HMGA2* contain target sequences of *miR-509-5p*. *HMGA2* is an EMT-promoting factor in several cancer types^27, 28^. We first checked the protein levels of these genes in *miR-509-5p* or *miR-1243* transfectants using western blot analysis and observed that their protein expression levels were decreased in *miR-509-5p* or *miR-1243* transfectants compared with *miR-NC* transfectants (Figs 2c,e and 3d). To determine whether these candidate target genes are directly regulated by *miR-509-5p* or *miR-1243*, we constructed reporter plasmids for each target gene (Supplementary Fig. S4a–c). We performed a luciferase reporter assay using plasmids containing the wild type or mutant 3′ UTR of *SMAD2*, *SMAD4*, *VIM*, *ZEB1* and *HMGA2*. We detected significant reductions in luciferase activity in wild-type constructs but not in mutant constructs for *SMAD2*, *SMAD4*, *VIM* and *HMGA2* (Fig. 3e). *miR-509-5p* could not bind to *ZEB1* (Supplementary Fig. S4b), indicating that *SMAD2* and *SMAD4* were direct targets of *miR-1243* and *VIM* and that *HMGA2* was a direct target of *miR-509-5p*. To evaluate the biological effects of knockdown of these miRNAs, we performed several biological experiments with KMP3 and CFPAC1 cells, which show higher expression of *miR-509-5p* and *miR-1243* than Panc1 cells. However, knockdown of each miRNA did not affect the EMT phenotype, cell growth, migration and invasion abilities (Supplementary Fig. S5).

### Suppression of HMGA2 inhibited cell migration and invasion abilities via an MET phenotype change

**Figure 4:**
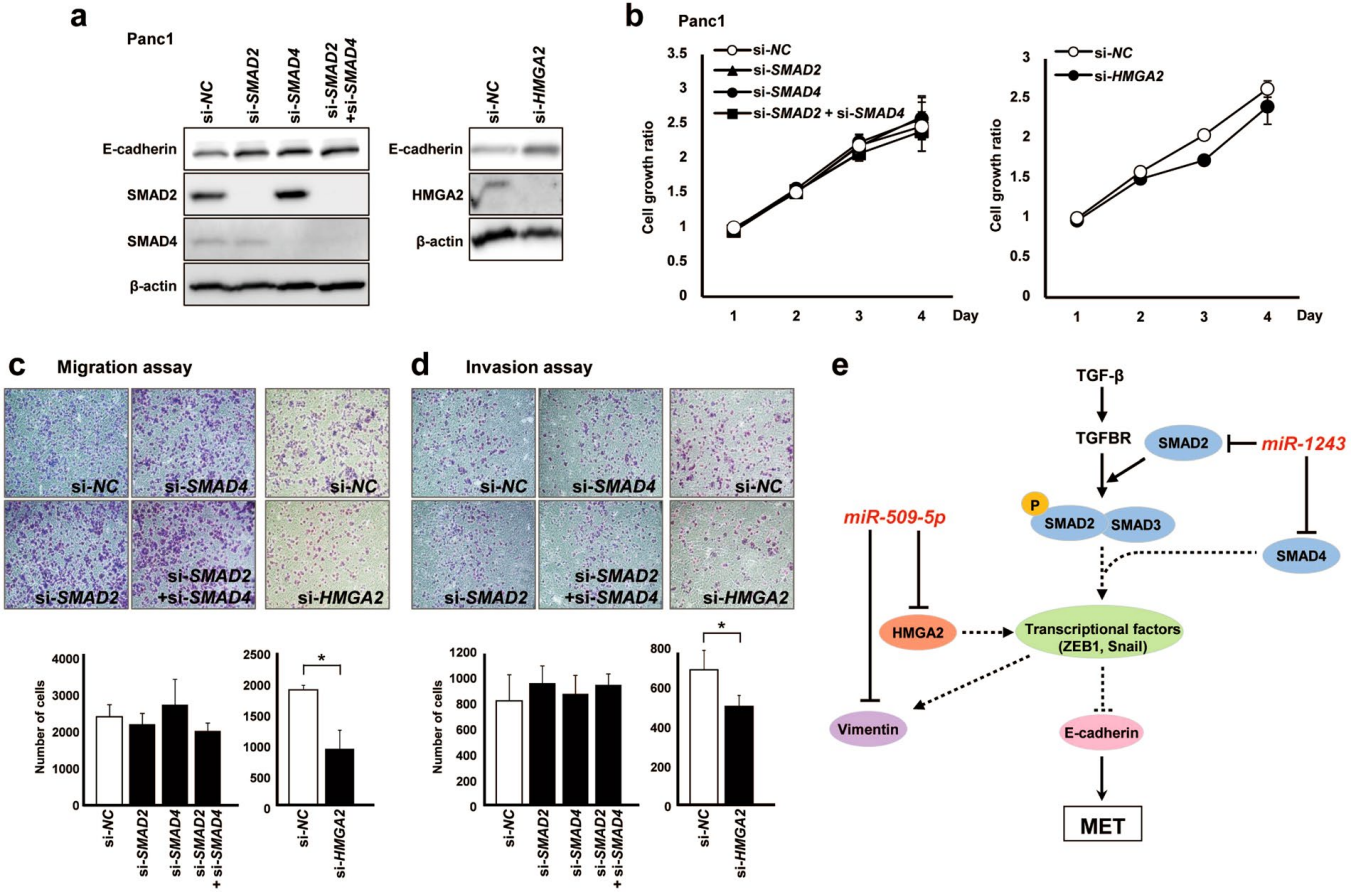
Suppression of *HMGA2* inhibits cell motility and invasion through an MET phenotype alteration, whereas these abilities are not affected by suppression of *SMADs*. (**a**) Knockdown of *SMAD2*, *SMAD4*, *SMAD2* plus *SMAD4* (left) and *HMGA2* (right) via specific siRNAs was confirmed by western blotting in Panc1 cells. The protein expression of these endogenous genes was downregulated by each specific siRNA compared with control siRNA. (**b**) The number of viable cells 24–72 hours after transfection of each siRNA was assessed by the WST-8 assay and is presented as the mean ± SD (bars) for triplicate experiments. (**c**,**d**) Transwell migration and invasion assays were performed in 24-well modified Boyden chambers without and with Matrigel, respectively. siRNA-transfected Panc1 cells (4 × 10^4^ cells per well [migration and invasion assay]) were transferred into the upper chamber, and the migrated or invaded cells on the lower surface of the filters were fixed, stained and counted after 24 hours of incubation. Experiments were performed in triplicate, and each data point represents the mean (bars, SD). Student’s *t*-test was used for statistical analysis, and asterisks represent *P* < 0.05 versus si*-NC* transfectants. (**e**) Model for the *miR-509-5p-* and *miR-1243*-mediated pathway in EMT.

To investigate whether these direct target genes of each miRNA induce an MET phenotype, we performed knockdown experiments using specific siRNAs against these direct target genes. We first confirmed that the expression of E-cadherin in si-*SMAD2*, si-*SMAD4* and si-*HMGA2* transfectants was increased compared with the si-*NC* transfectant in Panc1 cells, resulting in the induction of an MET phenotype (Fig. 4a). The cell growth rate was not affected by knockdown of these genes (Fig. 4b). We next investigated the cell migration and invasion abilities in each of the siRNA transfectants. Knockdown of *SMADs* did not reduce the migration and invasion abilities, suggesting that the inhibition of cell migration and invasion by *miR-1243* might be unrelated to the expression of *SMADs* (Fig. 4c,d). However, the altered MET phenotype could be induced by suppressing *SMADs*. In addition, we analyzed the effect of TGF-β in each of the siRNA-transfected cells and observed that suppressing *SMADs* reduced the effect of TGF-β (Supplementary Fig. S6). Although knockdown of *SMADs* did not affect cell proliferation under the addition of recombinant TGF-β, knockdown of *SMAD4* or *SMAD2*/*SMAD4* reduced the migration and invasion. Therefore, these results were consistent with the *miR-1243* overexpressed phenotype, indicating that *miR-1243* increased the expression of E-cadherin by regulating SMADs (Fig. 4e). On the other hand, *miR-509-5p* induced an MET phenotype by regulating HMGA2 (Fig. 4e).

### *miR-509-5p* and *miR-1243* enhanced the effect of gemcitabine

**Figure 5:**
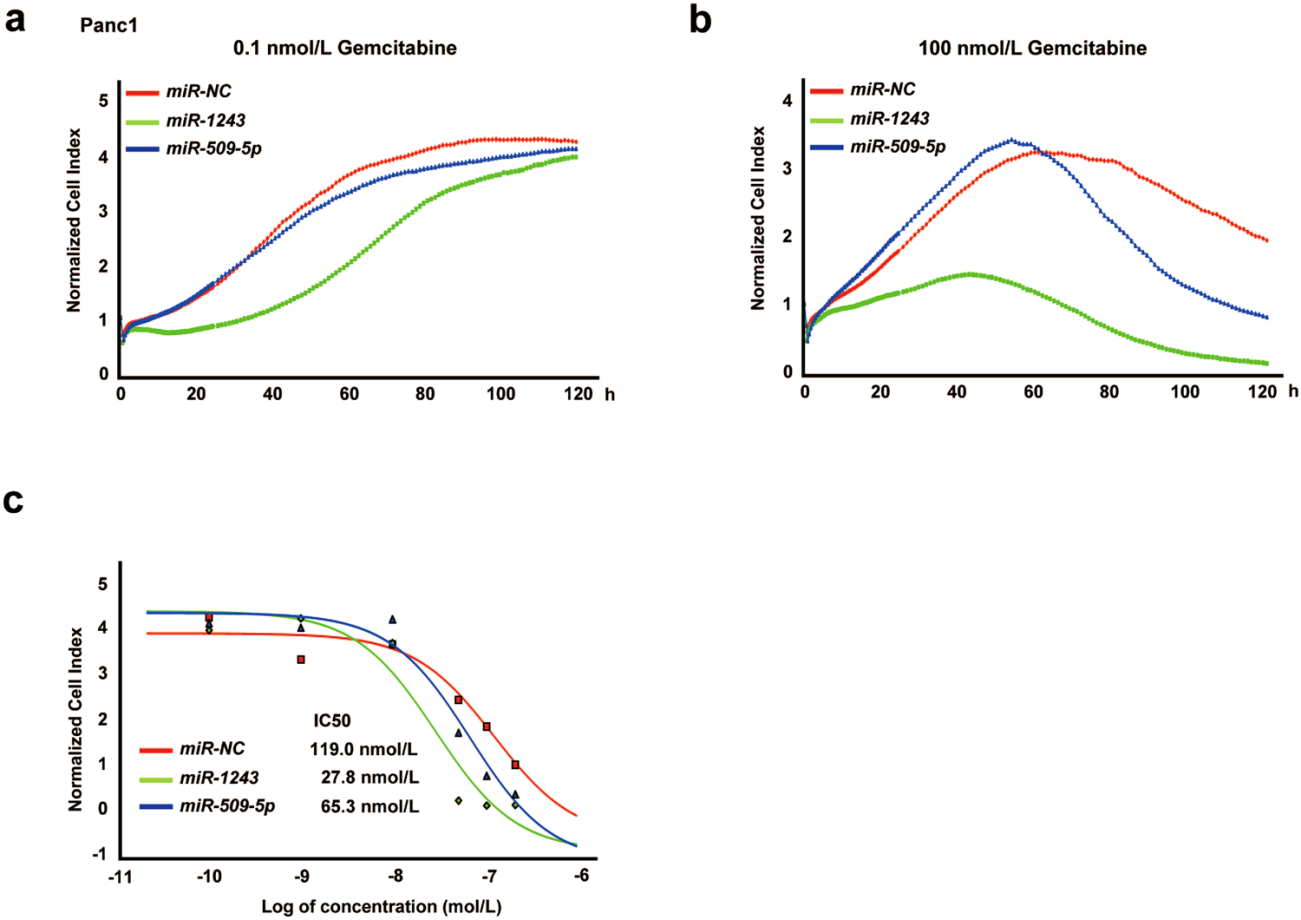
Overexpression of *miR-509-5p* and *miR-1243* enhances the effect of gemcitabine. (**a**,**b**) Effect of a combination of miRNA and gemcitabine on cell index curves (RTCA) in Panc1 cells. A total of 5 nmo/L of *miR-NC*, *miR-509-5p* or *miR-1243* was transfected, and cells were treated with gemcitabine (0.1, 1, 10, 50, 100 and 200 nmol/L) after 6 hours. Figure 5a shows 0.1 nmol/L gemcitabine-treated cells, and Fig. 5b shows 100 nmol/L gemcitabine-treated cells. Cell indexes were normalized with the last time point before treatment with gemcitabine. (**c**) Representative curves of the growth-suppressive effects at 120 hours following treatment with gemcitabine in cells transfected with *miR-NC*, *miR-509-5p* and *miR-1243*, and the IC50 in each miRNA transfectant.

Because EMT can contribute to the resistance of anti-cancer drugs^29^, we evaluated whether overexpression of these miRNAs increased their sensitivity to gemcitabine, an anti-cancer drug that is classified as an anti-metabolite. After treatment with several concentrations of gemcitabine (0.1, 1, 10, 50, 100 and 200 nmol/L) and PBS as a control in Panc1 cells, we measured real-time cell growth for 120 hours using the xCELLigence RTCA DP system (ACEA Biosciences, San Diego, CA, USA) (Fig. 5). In 0.1 nmol/L gemcitabine-treated cells, cell growth did not change in each miRNA transfectant at 120 hours post-treatment (Fig. 5a), whereas treating the *miR-509-5p* and *miR-1243* transfectants with 100 nmol/L gemcitabine showed an enhanced effect of gemcitabine compared with *miR-NC* transfectants (Fig. 5b). Moreover, we measured the IC50 of gemcitabine in each miRNA transfectant (*miR-NC*: 119 nmol/L, *miR-509-5p*: 65.3 nmol/L, *miR-1243*: 27.8 nmol/L) (Fig. 5c). Together, our data show that inducing an MET phenotype by *miR-509-5p* and *miR-1243* enhanced the cytotoxic effect of gemcitabine. Thus, a combination of each of these miRNAs and gemcitabine might be a novel therapy for pancreatic cancer.

### The expression level of *miR-509-5p* was associated with overall survival in patients with pancreatic cancer

**Figure 6:**
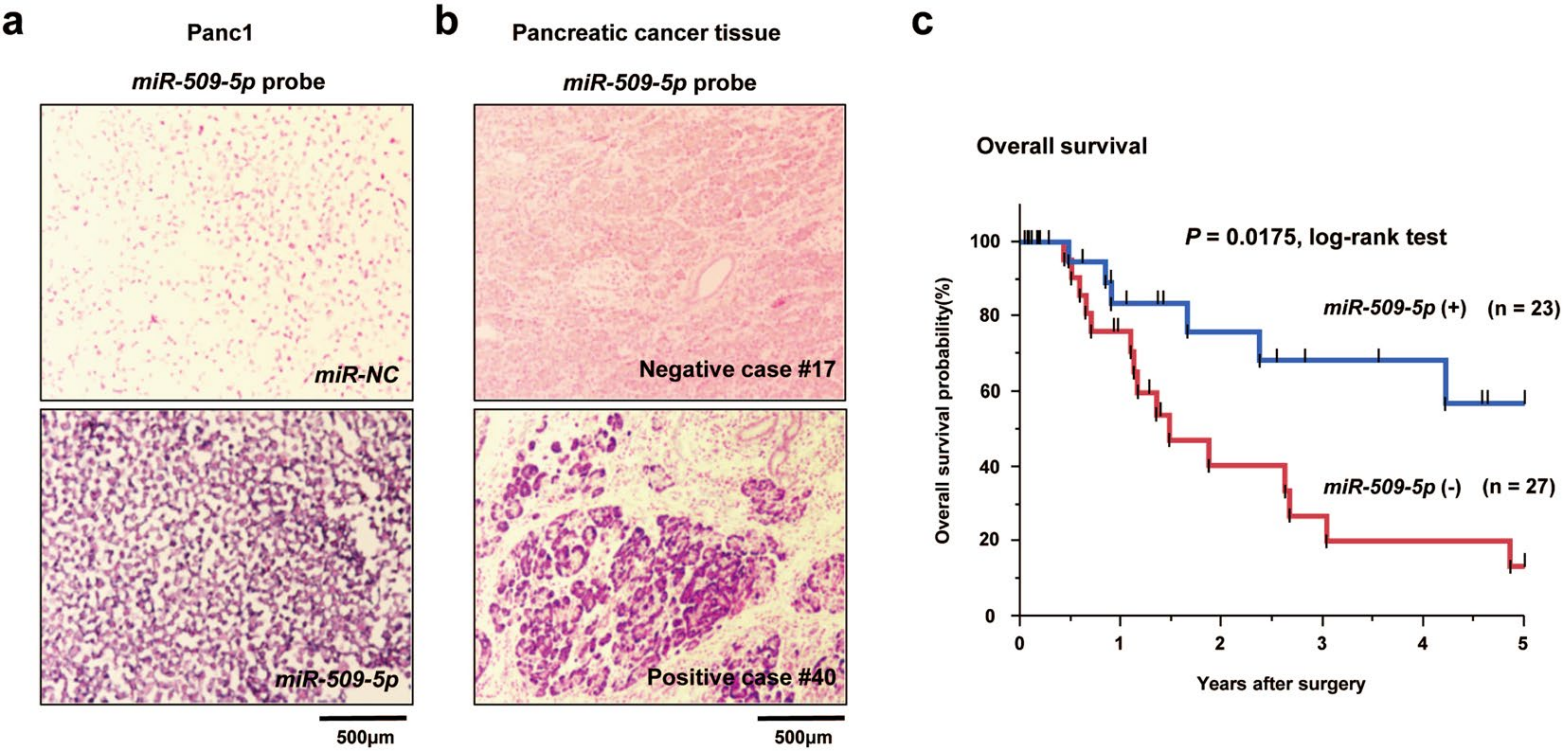
The expression of *miR-509-5p* is associated with overall survival in human PDAC. (**a**,**b**) Representative results of the *in situ* hybridization for *miR-509-5p*. (**a**) FFPE of Panc1 cells 24 hours after transfection with *miR-NC* (upper) and *miR-509-5p* (bottom). (**b**) Primary PDAC with negative staining (upper) and positive staining (bottom). (**c**) Kaplan-Meier curves for the overall survival rates of patients with primary PDAC. A lack of *miR-509-5p* expression in tumor cells was significantly associated with a worse overall survival (*P* = 0.0175, log-rank test).

To assess whether the expression of *miR-509-5p* and *miR*-*1243* is associated with disease outcome in patients with pancreatic cancer, we performed miRNA *in situ* hybridization (ISH) assay in 50 primary pancreatic tumors. We first prepared positive and negative controls by transfecting Panc1 cells with each miRNA and staining with a *miR-509-5p* or *miR-1243* miRNA probe (Figs 6a and S7a). After we confirmed that these miRNA probes were available, 50 primary pancreatic tumors were subjected to RNA ISH using each probe (Figs 6b and S7b). Kaplan-Meier survival estimates showed that low expression of *miR-509-5p* was significantly associated with worse overall survival in all cases (*P* = 0.0175, log-rank test) (Fig. 6c), whereas the expression of *miR-1243* was not related to the prognosis (*P* = 0.4842, log-rank test) (Supplementary Fig. S7c). Moreover, we examined the clinicopathological significance of the expression of *miR-509-5p* and *miR-1243* in 50 primary pancreatic tumors based on the RNA ISH staining pattern of each miRNA. We found that the expression of *miR-1243* was associated with the venous invasion category (*P* = 0.03) (Supplementary Table S2). In the Cox proportional hazard regression model (Supplementary Table S3), univariate analyses demonstrated that the expression of *miR-509-5p* and the lymphatic invasion category in the tumor-lymph node-metastases (TNM) classification were significantly associated with overall survival. In the multivariate analysis using a stepwise Cox regression procedure, the expression of *miR-509-5p* based on the TNM classification was identified as an independently selected predictive factor for overall survival in both forward and backward procedures (*P* = 0.0063). We also evaluated correlation between clinical features and each miRNA using the TCGA database, in which expression data of 1881 miRNAs on 178 pancreatic cancer samples have been stored. We examined the correlation of prognosis and expression of *miR-509-5p* and *miR-1243* using 141 samples excluding Stage IV, macroscopic residual tumors (R2) or unevaluable presence of tumors (RX), and tumor-types other than PDAC. However, Kaplan-Meier survival estimates showed the expression of *miR-509-5p* and *miR-1243* not to be associated with the prognosis (*P* = 0.6975, *P* = 0.3416, log-rank test, respectively), respectively (Supplementary Fig. S8).

## Discussion

In our previous study, we identified an EMT-suppressive miRNA, *miR-655*, using a function-based screening assay, which combined a cell-based reporter system with a miRNA library containing 470 miRNAs^20^. In the present study, we reliably observed that *miR-655* increased the expression of E-cadherin and induced an MET phenotype (Supplementary Table S1). The function-based screening assay performed in the current study also identified *miR-509-5p* and *miR-1243* as MET inducers. These two miRNAs were not explored in our previous study because these miRNAs were absent in the miRNA library V3 that was used. Several studies have demonstrated that *miR-509-5p* suppresses cell proliferation, invasion and migration of cervical cancer, hepatocellular carcinoma, renal carcinoma and breast cancer^30–32^. In addition, a recent study reported that *miR-509-5p* acts as an EMT-suppressive miRNA and inhibits cell migration and invasion by targeting *FOXM1* in non-small cell lung cancer^33^. However, only a few studies have focused on the correlation between EMT and *miR-509-5p*. Furthermore, the function of *miR-1243* has not yet been clarified.

We revealed that overexpression of *miR-509-5p* and *miR-1243* induced an MET phenotype by downregulating ZEB1 and Snail. However, our experiment using TGF-β showed different functional mechanisms between *miR-509-5p* and *miR-1243*: *miR-1243* depended on the SMAD signaling pathway, whereas *miR-509-5p* did not. Thus, these findings suggest that each miRNA has different target genes for the induction of the MET phenotype. We additionally evaluated the EMT marker genes, E-cadherin and vimentin, with two pancreatic cancer cell lines, SMAD4-intact SU.86.86 and SMAD4-homozygously-deleted BxPC3. Although overexpression of *miR-509-5p* reduced expression of vimentin, overexpression of *miR-509-5p* or *miR-1243* did not show up-regulation of E-cadherin in these cell lines (Supplementary Fig. S3). In general, pancreatic cancer cell lines, which have the EMT plasticity, are very few. In fact, since these cell lines have the epithelial phenotype, they were not induced the MET phenotype by each miRNA. Transfections of each miRNA inhibitor (anti-miRNA) did not affect EMT, cell growth, migration and invasion abilities in CFPAC1 and KMP3 cells (Supplementary Fig. S5). Furthermore, through TCGA database, almost PDAC shows low expression of *miR-509-5p* and *miR-1243*. Taken together, we concluded that inhibition of these miRNAs could not induce the EMT phenotype.

We next explored representative EMT-related genes from the Target Scan Human 7.1 database (http://www.targetscan.org/vert_71/) for the direct target-genes of each miRNA, and found that *miR-509-5p* regulates the expression of *VIM* and *HMGA2* by directly binding to their 3′ UTR regions. We next focused on the function of HMGA2 because it is an important regulator of cell growth, differentiation and EMT^27, 28^. HMGA2 is highly expressed in embryonic tissues and many malignant tumors, and overexpression of HMGA2 is associated with EMT, metastasis and poor prognosis in several cancers^34–36^. Because recent studies have revealed that some miRNAs, such as *miR-101*, *miR-221*, *miR-485-5p* and *let-7a*, inhibit EMT by targeting *HMGA2*, *HMGA2* is considered a key regulator of EMT^37–40^. In addition, the *Lin28b*-*let-7*-HMGA2 axis contributes to the maintenance of the stemness phenotype in hematopoietic cells^41^. Thus, our findings strongly suggest that *miR-509-5p* induces an MET phenotype by regulating *HMGA2*. In addition, low expression levels of *miR-509-5p* were significantly associated with worse overall survival in pancreatic cancer in our miRNA-ISH assay (Fig. 6). On the other hand, an additional evaluation of the correlation between *miR-509-5p* expression and the prognosis in patients with PDAC (n = 141 samples) in TCGA showed no significant value (*P* = 0.6975, log-rank test) in the Kaplan-Meier survival curve analysis (Supplementary Fig. S8). The statistical difference between TCGA and ours may be due to TCGA database not including Japanese PDAC tumors or a difference in the guidelines for the management of patients with pancreatic cancer between Japan and other countries such as the US and Europe.

We also explored direct target genes of *miR-1243* and identified *SMAD2* and *SMAD4* as direct target genes of *miR-1243*. Inhibition of *SMAD2* and/or *SMAD4* by *miR-1243* or their respective siRNAs increased the expression of E-cadherin. Although cell migration and invasion abilities were suppressed by *miR-1243*, knockdown of *SMAD2* and/or *SMAD4* did not affect their function. In general, *SMAD2* and *SMAD4* are well-known tumor suppressor genes^25, 26^. *SMAD4* is frequently inactivated by genomic alterations, such as deletion or mutation, contributing to cancer progression in pancreatic cancer, colorectal cancer and prostate cancer^42–45^. Furthermore, loss of *SMAD4* causes head and neck cancer in mice and promotes metastasis in human pancreatic cancer^46^. In this study, *SMAD2* and *SMAD4* inhibition showed no significant effect on cell proliferation of Panc1 cells. This finding was indeed consistent with previous studies in which *SMAD2* and *SMAD4* suppression did not induce cell growth in Panc1 cells *in vitro* ^47, 48^. *SMAD2* and *SMAD4* have an important role of the regulation of EMT/MET in TGF-β pathway. We also evaluated the cell growth, migration and invasion abilities by knockdown of *SMAD2* and/or *SMAD4* with treatment of recombinant TGF-β. Knockdown of *SMADs* with TGF-β did not affect cell growth, whereas TGF-β-induced migration and invasion were suppressed by knockdown of *SMAD4* or *SMAD2*/*SMAD4* (Supplementary Fig. S6), suggesting that the induction of MET phenotype by *miR-1243* might be relevant to the suppression of *SMAD2* and *SMAD4* which resulted in changing the cellular phenotype in part. However, in the present study, we could not identify direct target-genes of *miR-1243*, which regulate cell growth. Our findings suggest that the function of *miR-1243* in pancreatic cancer is likely dependent on the status of *SMAD2* and *SMAD4* as well as cancer-related genes other than *SMAD2* and *SMAD4*, further indicating the possible dependence on cellular-context manner^14, 49^. *miR-1243* might have some crucial role in TGF-β pathway via regulating the expression of SMAD2/4 in PDAC with wild-type SMAD2/4.

On the other hand, the expression of *miR-1243* in our miRNA-ISH assay was not associated with the prognosis in pancreatic cancer in contrast to *miR-509-5p*. Since there were only 50 PDAC samples in the present study, we may need to examine the relationship between the prognosis and expression of *miR-1243* in large scale with more number of cases.

Although EMT contributes to cancer progression, metastasis and drug resistance^6^, the mechanism by which it contributes to drug resistance is poorly understood. The role of miRNAs in regulating drug resistance through EMT in cancer also remains unclear. Our findings showed that overexpression of *miR-509-5p* and *miR-1243* increased the sensitivity of pancreatic cancer cells to gemcitabine. This result suggests that patients with high expression of these miRNAs in PDAC may have the sensitivity to gemcitabine compared to patients without the expression of these miRNAs. Thus, the expression status in these two miRNAs might predict gemcitabine efficacy in patients with pancreatic cancer. In addition, since the expression *miR-509-5p* was associated with the prognosis, *miR-509-5p* might be useful for a biomarker in the clinical setting of pancreatic cancer. Moreover, interestingly, recent studies have revealed that the inhibition of EMT in lung and pancreatic cancers increase drug sensitivity^18, 50^. Thus, combinatorial therapeutics with anti-cancer drugs and overexpression of *miR-509-5p* or *miR-1243* might serve as a novel cancer therapy that can overcome chemoresistance. Indeed, overexpression of *miR-509-5p* and *miR-1243* increased the sensitivity of pancreatic cancer cells to gemcitabine. We hypothesize that gemcitabine-induced cytotoxicity was increased via the induction of the MET phenotype through the downregulation of HMGA2, which in turn was caused by the overexpression of *miR-509-5p*. Because cell death could be induced by a single treatment of *miR-1243*, the combination of *miR-1243* and gemcitabine may have produced a synergistic effect on cell growth, i.e., the MET phenotype and other effects caused by *miR-1243* might increase the sensitivity to gemcitabine. Taken together, these results suggest that such combinatorial therapeutics might be useful for chemoresistant cancer.

In conclusion, we have established a cell-based reporter system to monitor the promoter activity of *CDH1* and identified *miR-509-5p* and *miR-1243* as EMT-suppressive miRNAs using this system. Overexpression of *miR-509-5p* and *miR-1243* markedly induced the MET phenotype and inhibited cell motility and invasion *in vitro* through the regulation of the target genes of each miRNA. In addition, *miR-509-5p* and *miR-1243* enhanced the effect of gemcitabine on cell growth. The expression level of *miR-509-5p* could predict prognosis in patients with pancreatic cancer. Our findings implicate the EMT-suppressive *miR-509-5p* and *miR-1243* as potential therapeutic targets for pancreatic cancer and suggest that *miR-509-5p* might be a prognostic biomarker. Importantly, our cell-based reporter system, which was used to identify EMT-related molecules, can be utilized with other libraries of cDNA, siRNA, shRNA and therapeutic compounds.

## Materials and Methods

### Cell Lines and Primary Tumor Samples

The pancreatic cancer cell lines, Panc1 and KMP3 were maintained in Dulbecco’s Modified Eagle Medium containing 10% fetal bovine serum (FBS). The pancreatic cancer cell line KP4-4, BxPC3, CFPAC1 and SU.86.86 were maintained in Roswell Park Memorial Institute medium 1640 containing 10% FBS. The human mammary carcinoma cell line MDA-MB-231 was purchased from the American Type Culture Collection (Manassas, VA, USA) and was maintained in L-15 medium containing 10% FBS. Pancreatic cancer cell lines were authenticated in our previous studies of array-CGH analyses^51^.

A total of 50 primary PDAC samples were obtained from patients with PDAC who underwent pancreatectomy at Kyoto Prefectural University of Medicine between 2000 and 2011. These samples were embedded in paraffin after 24 hours of formalin fixation. None of these patients underwent preoperative chemotherapy or radiotherapy, and none had metachronous multiple cancers in other organs. All samples were obtained with the informed consent of each patient after approvals by the local ethics committees of the Medical Research Institute and Faculty of Medicine in Tokyo Medical and Dental University (approval number: 2015-001) and Kyoto Prefectural University of Medicine (approval number: ERB-C-67-2). The methods were carried out in accordance with the approved guidelines and regulations.

### Screening system using the promoterless expression vector pZsGreen1

The two stable clones, PEcadZsG-Panc1 #1 and #2 cells, were established by the limiting dilution method after transfection of the reporter construct into Panc1 cells. First, PEcadZsG-Panc1 #1 (5 × 10^3^ cells per well) and #2 (3 × 10^3^ cells per well) were seeded on 96-well plates. After 24 hours, each clone was transfected in duplicate with one of 1090 dsRNAs from the Pre-miRTM miRNA Precursor Library-Human V15 (Thermo Fisher Scientific, CA, USA) or with a negative control RNA, using an RNA concentration of 10 nmol/L. After 72 hours, the fluorescence intensity of the ZsGreen1 protein was measured by ARVO mx (Perkin Elmer, MA, USA). At the same time, cell survival was assessed by the crystal violet staining assay. The relative fluorescence intensity was calculated by the formula: fluorescence intensity/cell survival rate^22^.

### Transfection of miRNAs, siRNAs and miRNA inhibitors and treatment with TGF-β

The dsRNA mimicking mature human miRNA for *miR-200c* (MC11714), *miR-509-5p* (MC13068), *miR-660* (MC11216), *miR-1243* (MC13161) and negative control miRNA (negative control #1) were purchased from Thermo Fisher Scientific. The siRNA for *Smad2* (L-003561-00), *Smad4* (L-003902-00), *HMGA2* (L-013495-00), and negative control siRNAs (D-001810-05) were purchased from GE Healthcare (Buckinghamshire, UK). The miRNA inhibitors for anti-*miR-509-5p* (MH13068), anti-*miR-1243* (MH13161) and negative control anti-miRNA (negative control #1) were purchased from Thermo Fisher Scientific. miRNAs, siRNAs and miRNA inhibitors were transfected individually into cells at the indicated concentrations using Lipofectamine RNAiMAX (Thermo Fisher Scientific) according to the manufacturer’s instructions. After each miRNA transfectant was treated with TGF-β (5 ng/ml), the expression of E-cadherin was evaluated by western blot analysis.

### Quantitative RT–PCR (qRT-PCR)

Total RNA was extracted using TRIsure reagent (BIOLINE, London, UK) according to standard procedures. For miRNA, total RNA was reverse transcribed using Taqman Reverse Transcription Kit followed by qRT-PCR performed using Custom Taqman miRNA Assays kit (Applied Biosystems). The miRNA expression was normalized to endogenous control RNU6B. Single-stranded cDNA generated from the total RNA was amplified with a gene-specific primer set. Gene expression was normalized to the housekeeping gene *glyceraldehyde-3-phosphate dehydrogenase* (*GAPDH*). The qRT-PCR was performed using an ABI PRISM 7500 sequence detection System (Applied Biosystems, Foster City, CA, USA) according to the manufacturer’s instructions. The following primers were used for the Taqman assay (Thermo Fisher Scientific): human *miR-509-5p* (002235), *miR-1243* (002854), *RNU6B* (001093), *CDH1* (Hs01023894_m1), *VIM* (Hs00958111_m1) and *GAPDH* (Hs02758991_g1).

### Western blotting and immunofluorescent staining

The following primary antibodies were used for western blotting or immunofluorescent staining: anti-E-Cadherin (#610181) (BD Biosciences, Franklin Lakes, NJ, USA), anti-HMGA2 (#8179), anti-Snail (#3879), anti-TCF8/ZEB1 (#3396), anti-ZO-1 (#5406) (Cell Signaling Technology, Danvers, MA, USA), anti-Vimentin Ab-2 (Clone V9) (Thermo Fisher Scientific), anti-Smad2 (ab33875) (Abcam, Cambridge, UK), anti-β-actin (Sigma, St. Louis, MO, USA) and anti-Smad4 (sc-7966) (Santa Cruz Biotechnology, Santa Cruz, CA, USA). Western blotting and immunofluorescent staining were performed as described elsewhere^52^.

### Cell survival, migration and invasion assays

The number of living cells at various time points after transfection was assessed by a colorimetric water-soluble tetrazolium salt (WST) assay or crystal violet staining assay, as described elsewhere^53^. Transwell migration and invasion assays were performed in 24-well modified chambers precoated without (migration) or with (invasion) Matrigel (BD BioCoat, BD Biosciences), as described elsewhere^54^. Panc1, CFPAC1 and KMP3 cells (4 × 10^4^ per well) in serum-free medium were transferred into the upper chamber. After 24 hours of incubation, the cells that migrate to the lower chamber, which contained 10% FBS as a chemoattractant, were fixed, stained with the Diff-Quik stain (Sysmex, Kobe, Japan) and counted in five random fields.

### Luciferase activity assay

Luciferase reporter plasmids were made by inserting the 3′-UTR of *Smad2*, *Smad4*, *VIM*, *HMGA2* and *ZEB1* downstream of the luciferase gene within a pmirGLO Dual-Luciferase miRNA Target Expression Vector (Promega, Madison, WI, USA). All site-specific mutations used the GeneTailor site-directed mutagenesis system (Thermo Fisher Scientific). Luciferase reporter plasmid or control plasmid (pmirGLO) was transfected into Panc1 cells using Lipofectamine 2000 (Thermo Fisher Scientific), and 10 nmol/L of miRNA (*miR-NC*, *miR*-*509-5p* or *miR-1243*) was also transfected 6 hours later. After two days, Firefly and Renilla luciferase activities were measured using the Dual-Luciferase Reporter Assay System (Promega), and relative luciferase activity was calculated by normalizing the Firefly luciferase reading with its corresponding internal Renilla luciferase control.

### Cytotoxicity study

Panc1 cells (5 × 10^3^) were seeded in wells of the E-Plate 16 (ACEA Biosciences, San Diego, CA, USA). Approximately 24 hours later, 5 nmol/L of each miRNA (*miR-NC*, *miR-509-5p* or *miR-1243*) was transfected into Panc1 cells. Six hours later, these transfectants were treated with gemcitabine (0.1, 1, 10, 50, 100 and 200 nmol/L) and PBS. Cell-electrode impedance was monitored using the xCELLigence RTCA DP system (ACEA Biosciences) to produce time-dependent cell response dynamic curves. Data were collected every 10 min after treatment with gemcitabine for the first four hours, every 15 min for the next 20 hours, and then every 1 hour for an additional 4 days.

### miRNA-ISH assay

The ISH assay was performed on formalin-fixed and paraffin-embedded (FFPE) tissue sections according to the manufacturer’s instructions (miRCURY LNA microRNA ISH Optimization Kit; Exiqon Inc., Vedbaek, Denmark). Briefly, the sections were deparaffinized in xylene, rehydrated with graded ethanol and incubated with proteinase-K for 10 min at 37 °C. Then, the sections were hybridized with the *miR-509-5p* and *miR-1243* double-digoxigenin (DIG)-labeled LNA probes for 1 hour at 55 °C and were washed stringently prior to incubation with blocking for 15 min and probing with specific anti-DIG antibody directly conjugated with alkaline phosphatase. Finally, the sections were counterstained with nuclear red. We classified samples stained even a little as each miRNA-positive groups and samples with no stain as each miRNA-negative groups.

### Public data sets

To explore the generality of the miRNA expression and clinical features among pancreatic cancer, we examined the publicity dataset from TCGA (http://cancergenome.nih.gov) retrieved on 20th February 2017. We took the primary pancreatic cancer data (TCGA-PAAD) from the TCGA data set, which included mRNA data on 178 samples and 1881 miRNAs, and examined correlation of prognosis and expression of *miR-509-5p* and *miR-1243* using 141 samples excluding Stage IV, macroscopic residual tumors (R2) or unevaluable presence of tumors (RX), and tumor-types other than PDAC. Expression of *miR509-5p* was taken as the sum of expression of *miR-509-1* and *miR-509-2*.

### Statistical analysis

The association between clinicopathological characteristics and the status of *miR-509-5p* or *miR-1243* expression in patients with PDAC was evaluated with the χ^2^ or Fisher’s exact test. In Kaplan-Meier curves, differences between subgroups were tested with the log-rank test. Univariate and multivariate survival analyses were performed using the likelihood ratio test of the stratified Cox proportional hazards model. Differences between subgroups were tested with the Student’s *t*-test and considered significant at *P* < 0.05.

## Electronic supplementary material

Supplementary Information

Supplementary Table S1a

Supplementary Table S1b

Supplementary Table S2

Supplementary Table S3
