# Supplementary material for: miR-509-5p and miR-1243 increase the sensitivity to gemcitabine by inhibiting epithelial-mesenchymal transition in pancreatic cancer
Source: Sci Rep. 2017 Jun 21;7:4002. doi: 10.1038/s41598-017-04191-w (PMC5479822; doi:10.1038/s41598-017-04191-w)

miR-509-5p and *miR-1243* increase the sensitivity to gemcitabine by inhibiting epithelial-mesenchymal transition in pancreatic cancer

Hidekazu Hiramoto, M.D.[1,3], Tomoki Muramatsu, Ph.D.[1], Daisuke Ichikawa, M.D., Ph.D.[3,6], Kousuke Tanimoto, Ph.D.[4], Satoru Yasukawa, M.D., Ph.D.[5], Eigo Otsuji, M.D., Ph.D.[3] and Johji Inazawa, M.D., Ph.D.[1,2]

[1]Department of Molecular Cytogenetics, Medical Research Institute, Tokyo Medical and Dental University, Tokyo, Japan. [2]Bioresource Research Center, Tokyo Medical and Dental University, Tokyo, Japan. [3]Department of Digestive Surgery, Graduate School of Medical Science, Kyoto Prefectural University of Medicine, Kyoto, Japan. [4]Genome Laboratory, Medical Research Institute, Tokyo Medical and Dental University, Tokyo, Japan. [5]Department of Pathology, Kyoto Prefectural University of Medicine, Kyoto, Japan. [6]First Department of Surgery, Faculty of Medicine, University of Yamanashi, Yamanashi, Japan.

Supplementary Information

Supplementary Figure S1. Map of the promoter region of the *CDH1*/E-cadherin gene and reporter construct for the establishment of a cell-based reporter system.

Supplementary Figure S2. Expression of *miR-509-5p* and *-1243* in 24 pancreatic cancer cell lines and normal pancreatic tissue.

Supplementary Figure S3. *miR-509-5p* and *miR-1243* did not induce an MET phenotype in a couple of pancreatic cancer cell lines.

Supplementary Figure S4. The design of each reporter construct for identification of direct target genes in each miRNA.

Supplementary Figure S5. Knockdown of each miRNA did not affect EMT phenotype, cell proliferation, motility and invasion.

Supplementary Figure S6. Suppression of SMADs reduces the effect of TGF-β.

Supplementary Figure S7. The expression of *miR-1243* is not associated with overall survival.

Supplementary Figure S8. The expression of *miR-509-5p* and *miR-1243* is not correlated with overall survival in a corresponding cohort of 141 patients with pancreatic ductal adenocarcinoma (PDCA) in TCGA database.

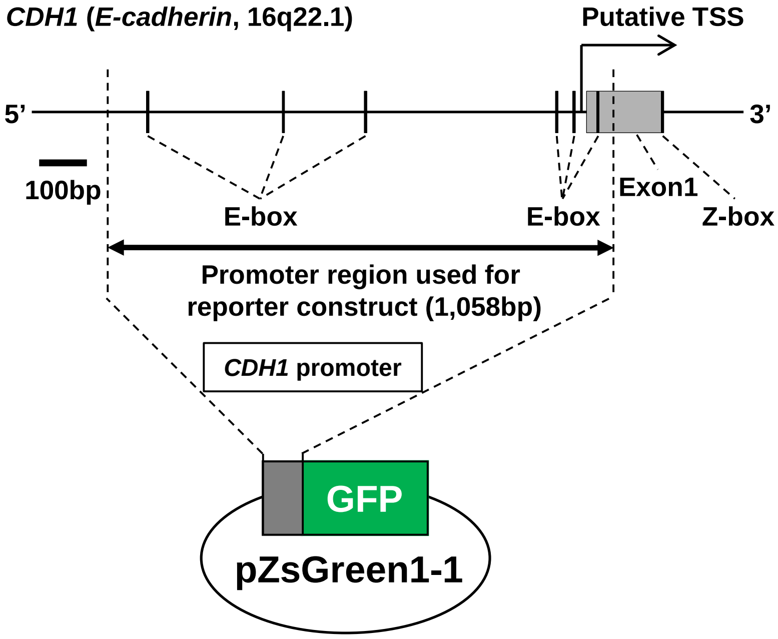

Supplementary Figure S1. Map of the promoter region of the *CDH1*/E-cadherin gene and reporter construct for the establishment of a cell-based reporter system.

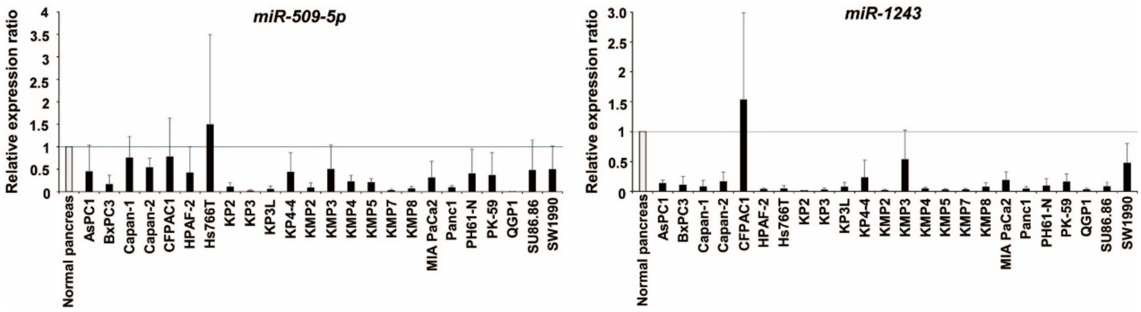

Supplementary Figure S2. Expression of *miR-509-5p* and *-1243* in 24 pancreatic cancer cell lines and normal pancreatic tissue.

The expression of *miR-509-5p* (left) and *miR-1243* (right) in a panel of 24 pancreatic cancer cell lines using qRT-PCR. Relative expression levels of *miR-509-5p* and *miR-1243* transcripts were quantified in comparison to RNU6B. Bar graphs show the ratio of the expression level in these cell lines to that in normal pancreatic tissue (bars, SD).

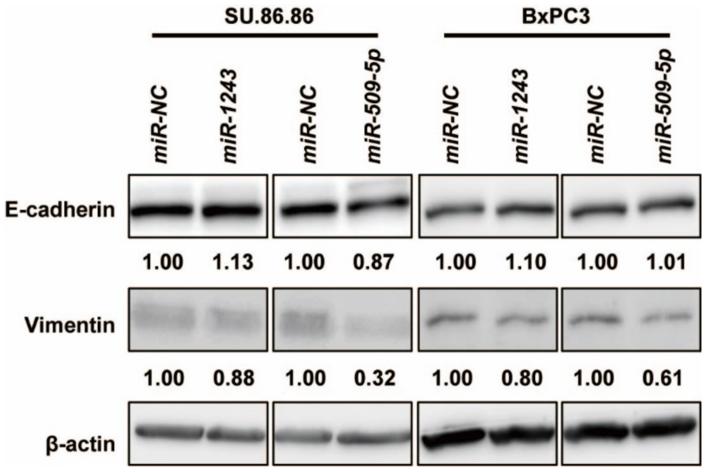

Supplementary Figure S3. *miR-509-5p* and *miR-1243* did not induce an MET phenotype in a couple of pancreatic cancer cell lines.

Western blot analysis of E-cadherin and Vimentin protein levels in SU.86.86 and BxPC3 cells 72 hours after transfection of 10 nmol/L of *miR-NC*, *miR-509-5p* or *miR-1243*.

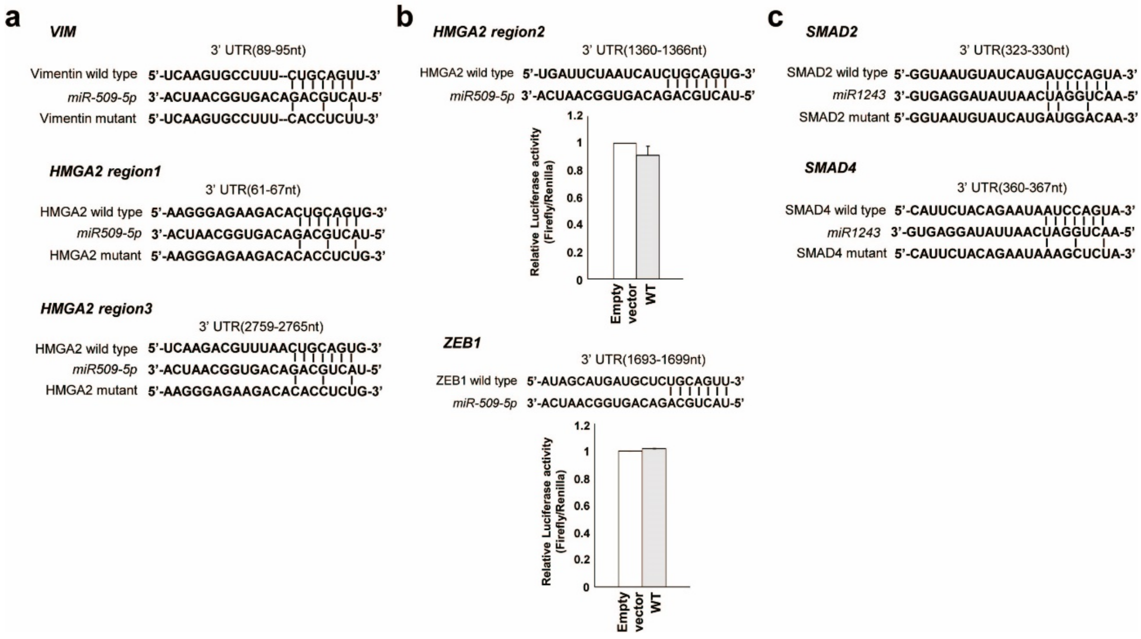

Supplementary Figure S4. The design of each reporter construct for identification of direct target genes in each miRNA.

(**a** and **b**) The putative binding sites of *miR-509-5p* in the 3'-UTR region of *VIM*, *HMGA2* and *ZEB1*. These sites were analyzed using TargetScan Human 7.1. (**b**) Results of luciferase reporter assay of *HMGA2* and *ZEB1*. (**c**) The putative binding sites of *miR-1243* in the 3'-UTR region of *SMAD2* and *SMAD4*.

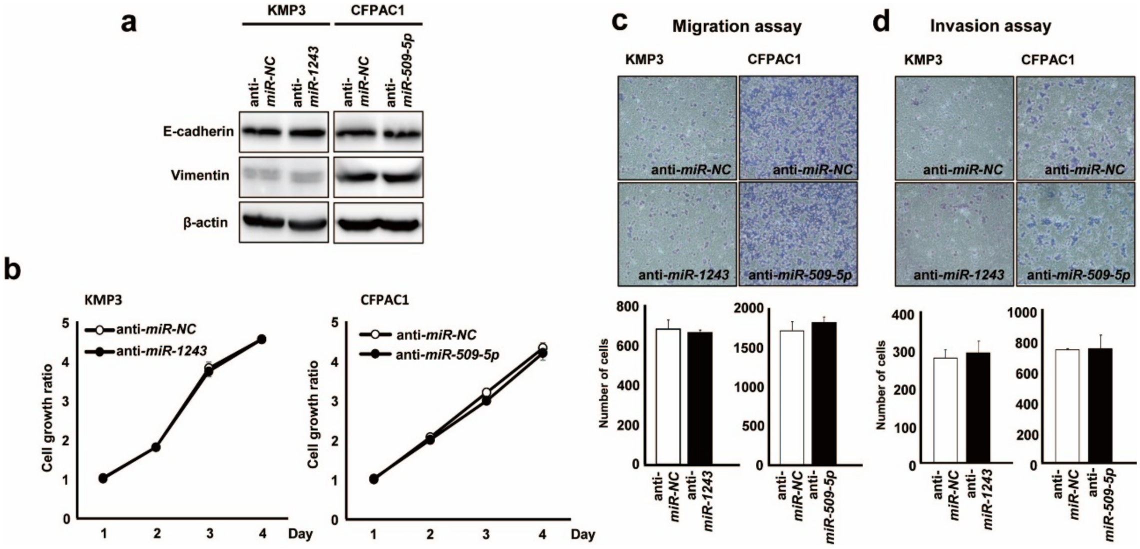

Supplementary Figure S5. Knockdown of each miRNA did not affect EMT phenotype, cell proliferation, motility and invasion.

(a) The results of western blotting of E-cadherin and Vimentin in KMP3 and CFPAC1 cells 72 hours after transfection with anti-*miR-NC*, anti-*miR-1243* or anti-*miR-NC* and anti-*miR-509-5p*. (b) The number of viable cells 24-72 hours after transfection of each 40 nmol/L of anti-miRNA was assessed by the WST-8 assay. Each data point represents the mean of triplicate experiments (bars, SD). (c and d) Transwell migration and invasion assays were performed in 24-well modified Boyden chambers without and with Matrigel, respectively. Experiments were performed in triplicate, and each data point represents the mean (bars, SD).

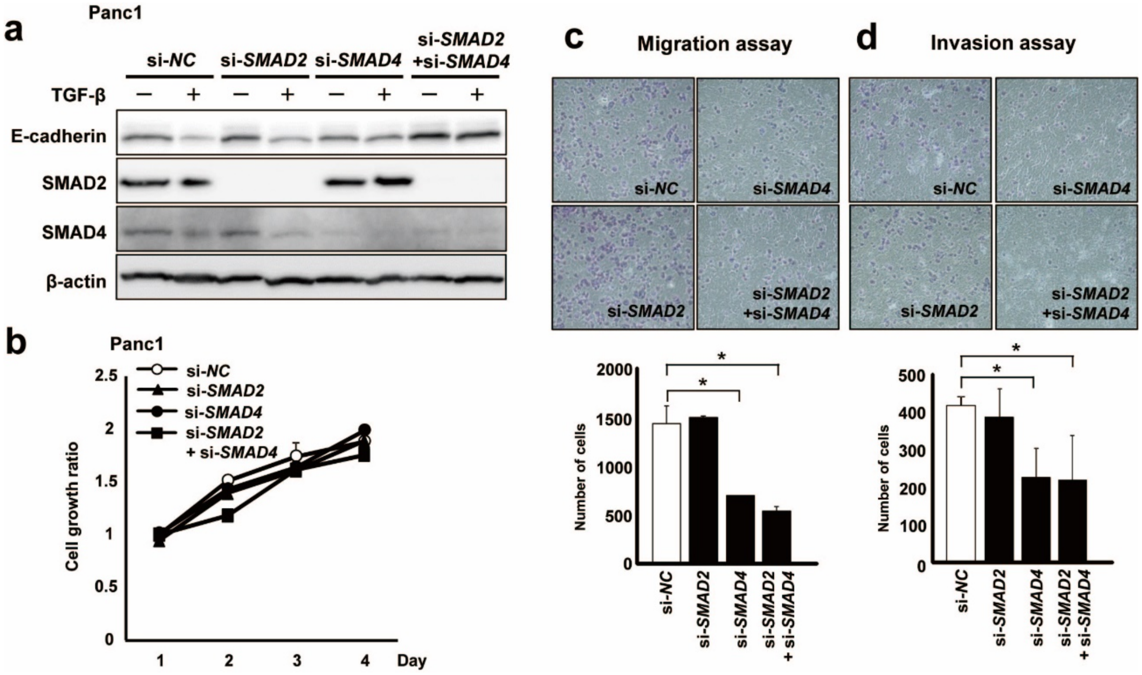

Supplementary Figure S6. Suppression of *SMADs* reduces the effect of TGF-β.

(a) The results of western blotting of E-cadherin, SMAD2 and SMAD4 in Panc1 cells 48 hours after treatment with or without TGF-β (5 ng/ml) and transfection with si-*NC*, si-*SMAD2*, si-*SMAD4* and si-*SMAD2* plus si-*SMAD4*. (b) The number of viable cells 24-72 hours after transfection of each 20 nmol/L of siRNA was assessed by the WST-8 assay. These transfectants were treated with TGF-β (5 ng/ml) 24hours after each siRNA transfection. Each data point represents the mean of triplicate experiments (bars, SD). (c and d) Transwell migration and invasion assays were performed in 24-well modified Boyden chambers without and with Matrigel, respectively. siRNA-transfected Panc1 cells (2×10^4 cells per well [migration and invasion assay]) were transferred into the upper chamber, and the migrated or invaded cells on the lower surface of the filters were fixed, stained and counted after 24 hours of incubation. Experiments were performed in triplicate, and each data point represents the mean (bars, SD).

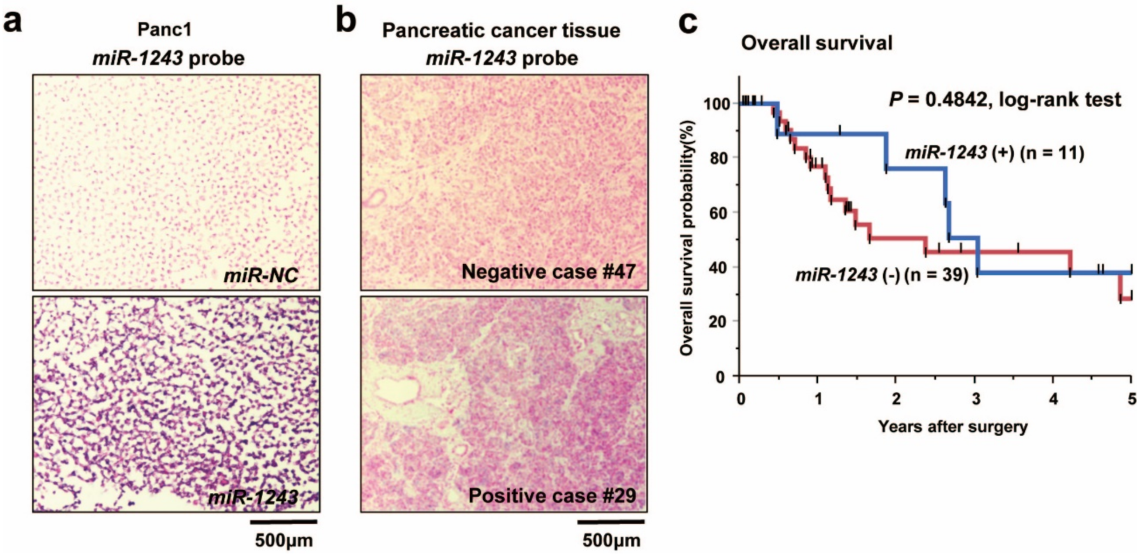

Supplementary Figure S7. The expression of *miR-1243* is not associated with overall survival.

(a and b) Representative results of in situ hybridization assay of *miR-1243*. (A) FFPE of Panc1 cells, 24 hours after transfection of *miR-NC* (upper) and *miR-1243* (bottom). (b) Primary PDAC with negative staining (upper) and positive staining (bottom). (c) Kaplan-Meier curves for overall survival rates of patients with primary PDAC. The expression of *miR-1243* in tumor cells was not associated with overall survival (P = 0.4842, log-rank test).

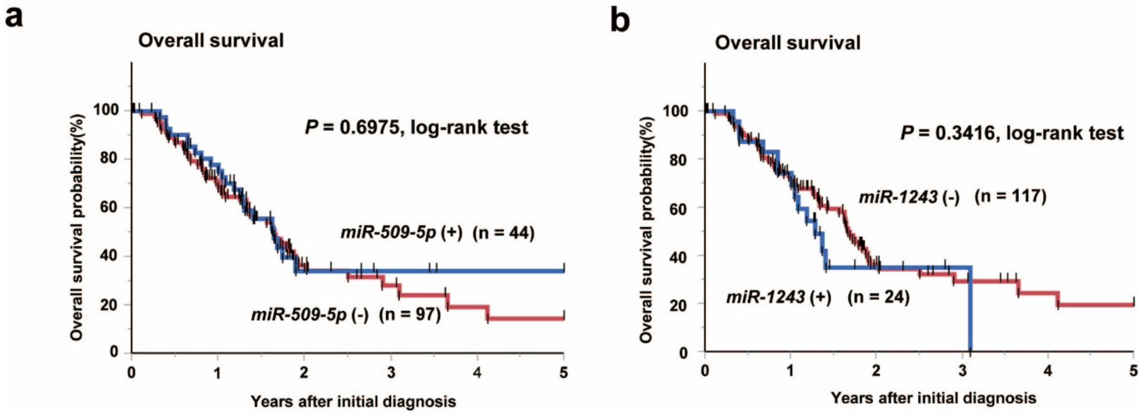

Supplementary Figure S8. The expression of *miR-509-5p* and *miR-1243* is not correlated with overall survival in a corresponding cohort of 141 patients with pancreatic ductal adenocarcinoma (PDCA) in TCGA database.

(a and b) Kaplan-Meier curves for overall survival rates of patients with primary PDAC in TCGA data. The expression of *miR-509-5p* (left) and *miR-1243* (right) in tumor cells was not correlated with overall survival (P = 0.6975, P = 0.3416, log-rank test, respectively).

Figure 1d

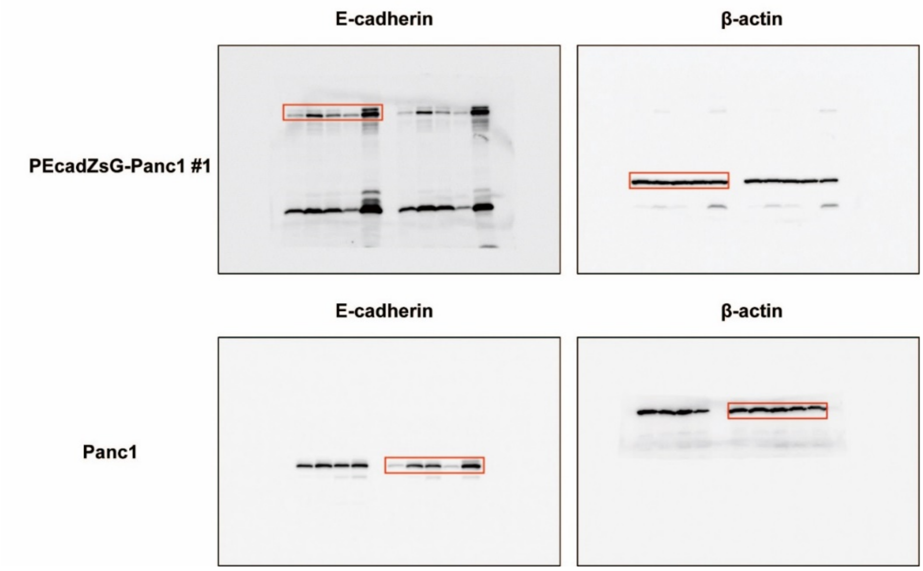

Figure 2c

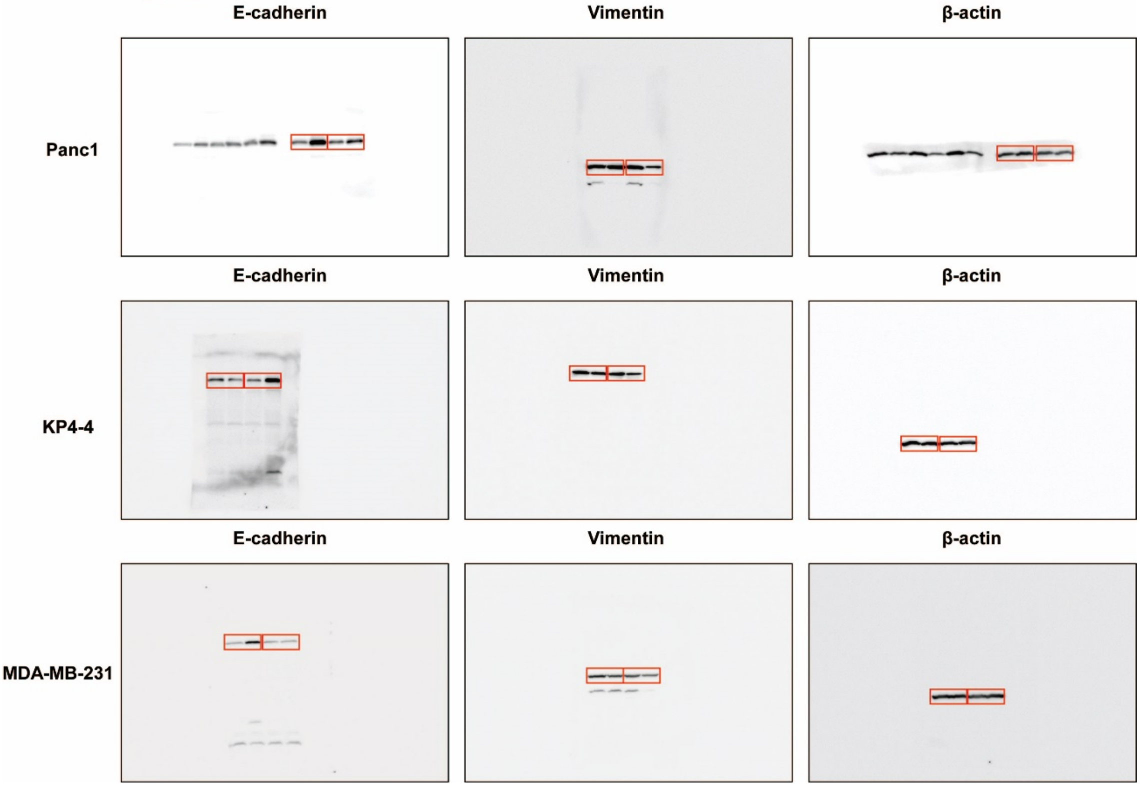

Figure 2d

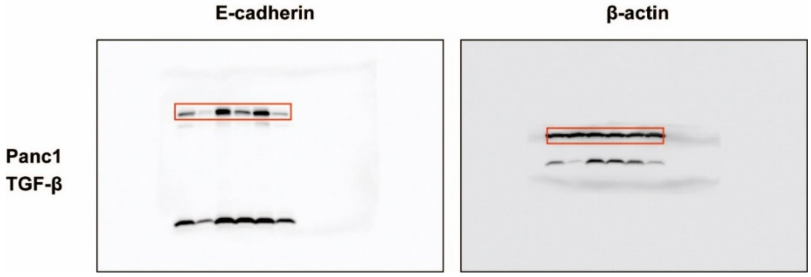

Figure 2e

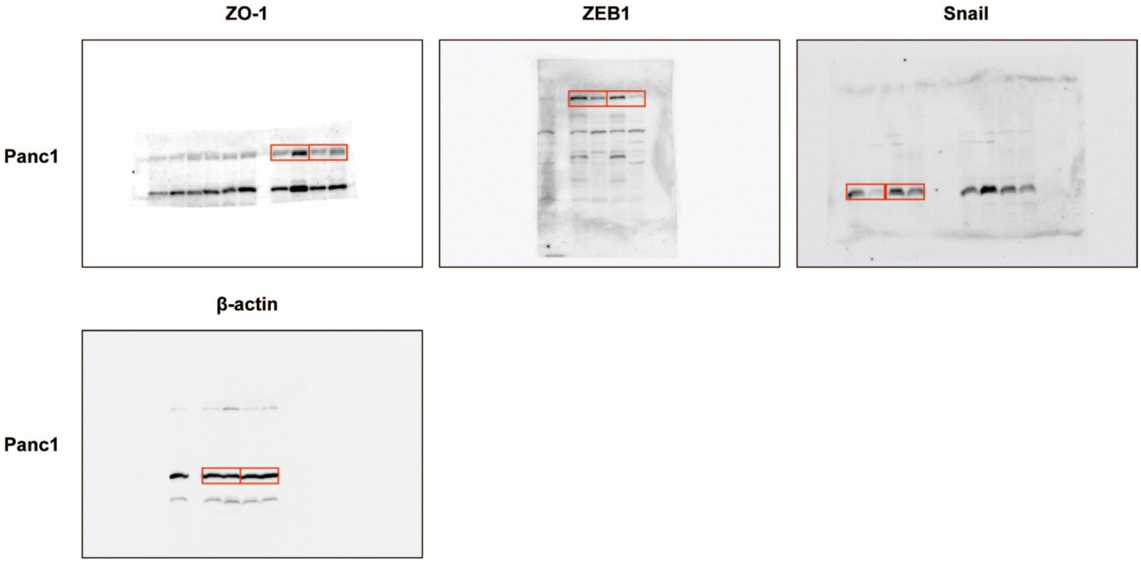

Figure 3d

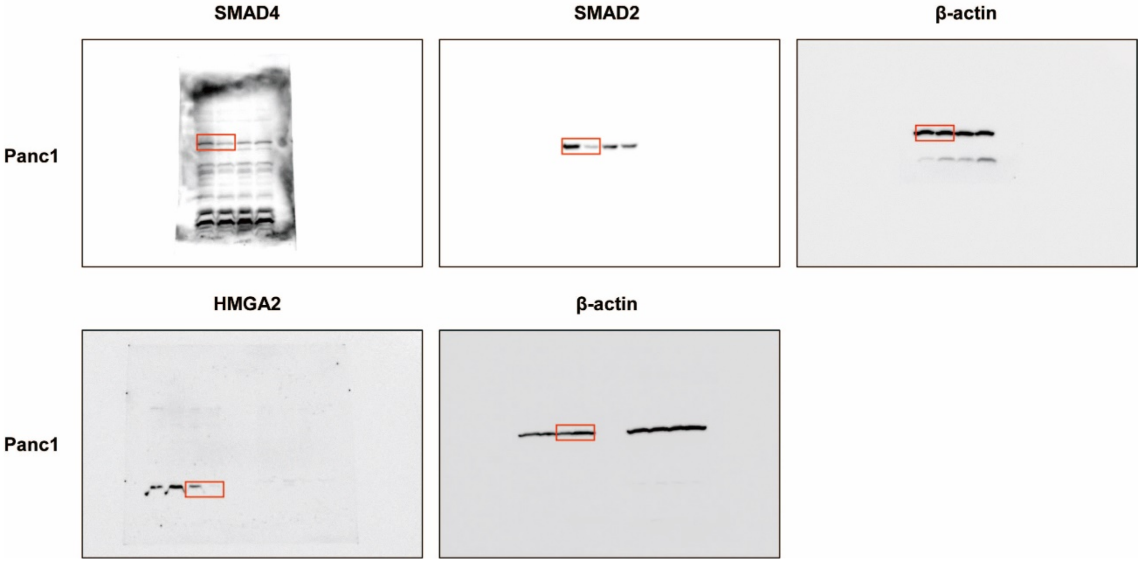

Figure 4a

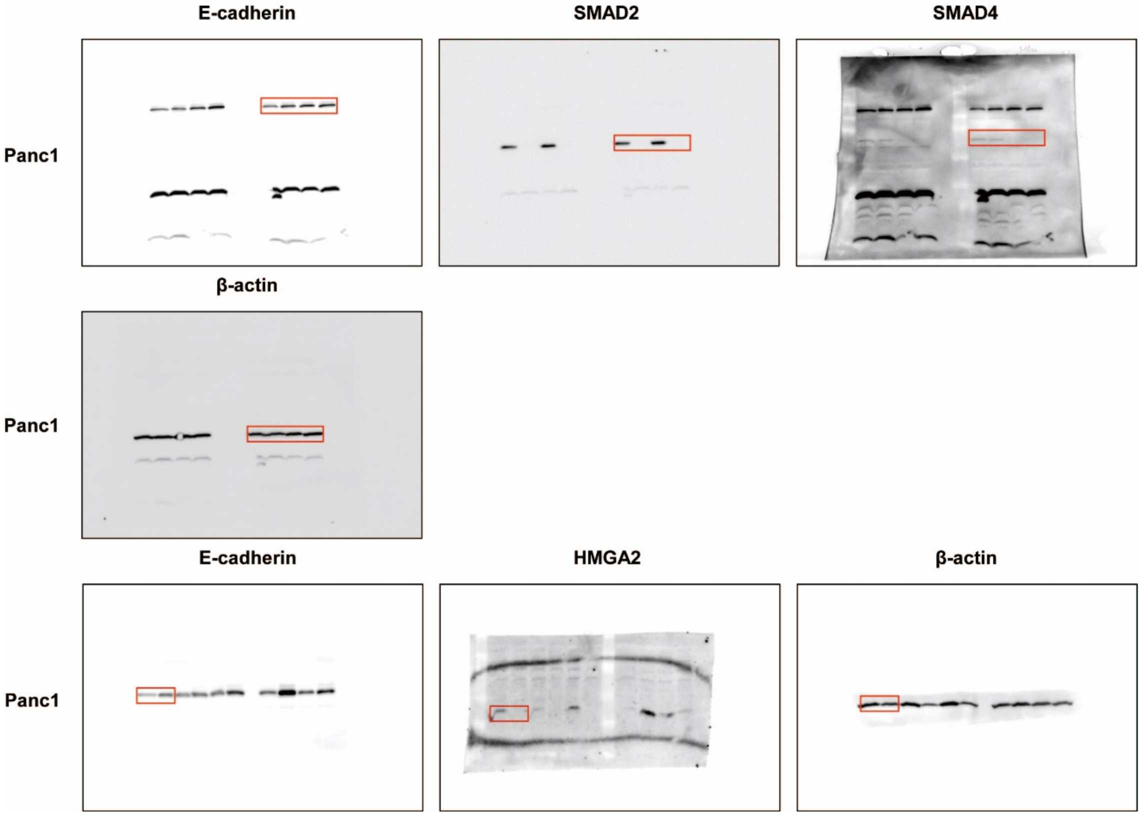
E-cadherin
SMAD2
SMAD4
Panc1
β-actin
Panc1
E-cadherin
HMGA2
β-actin
Panc1

Supplementary Figure S3

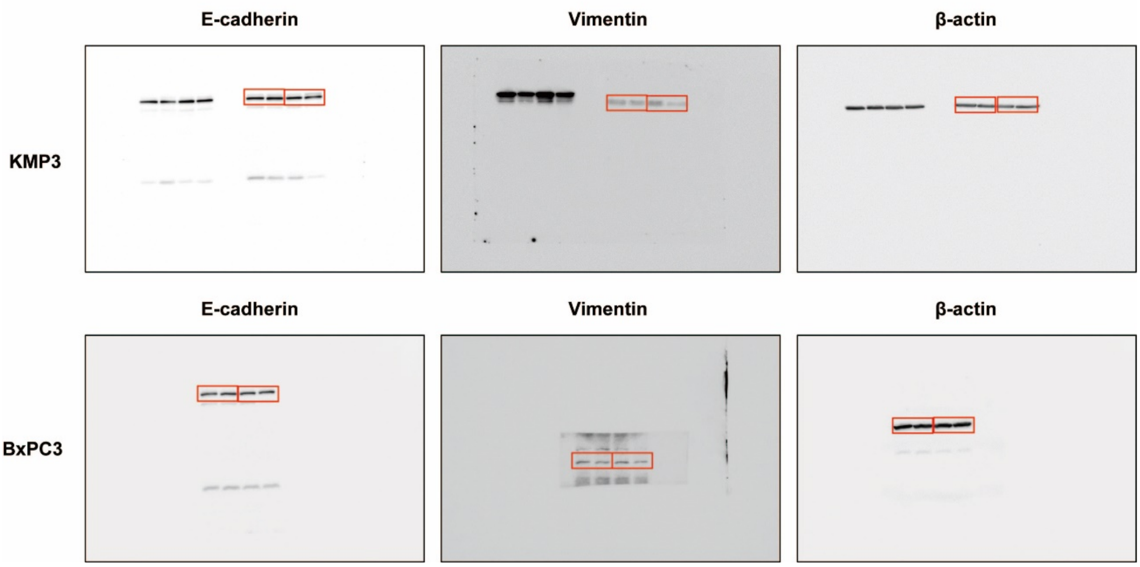

Supplementary Figure S5

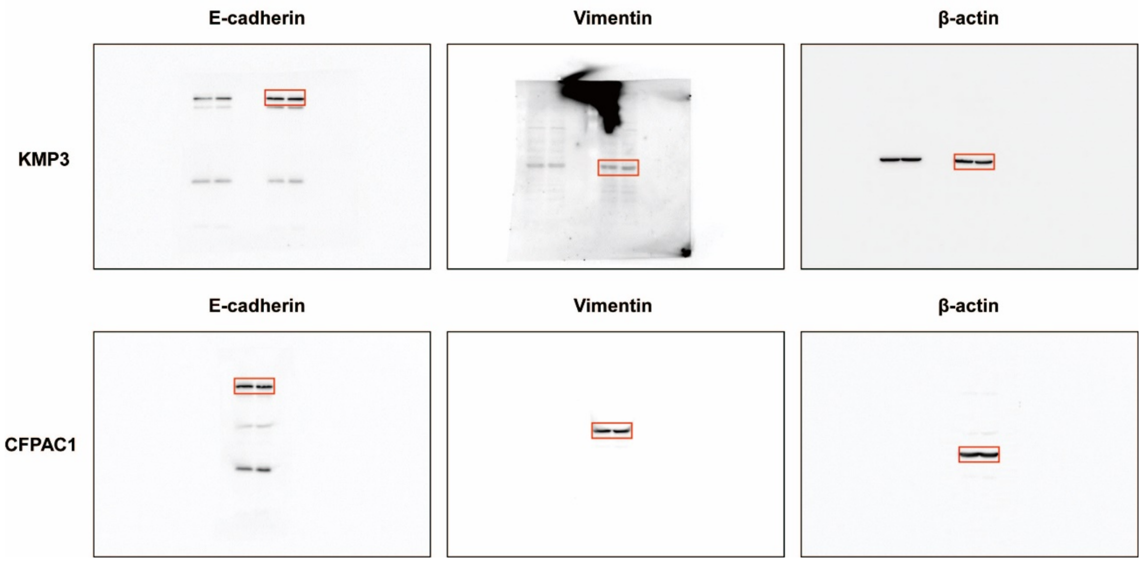

Supplementary Figure S6

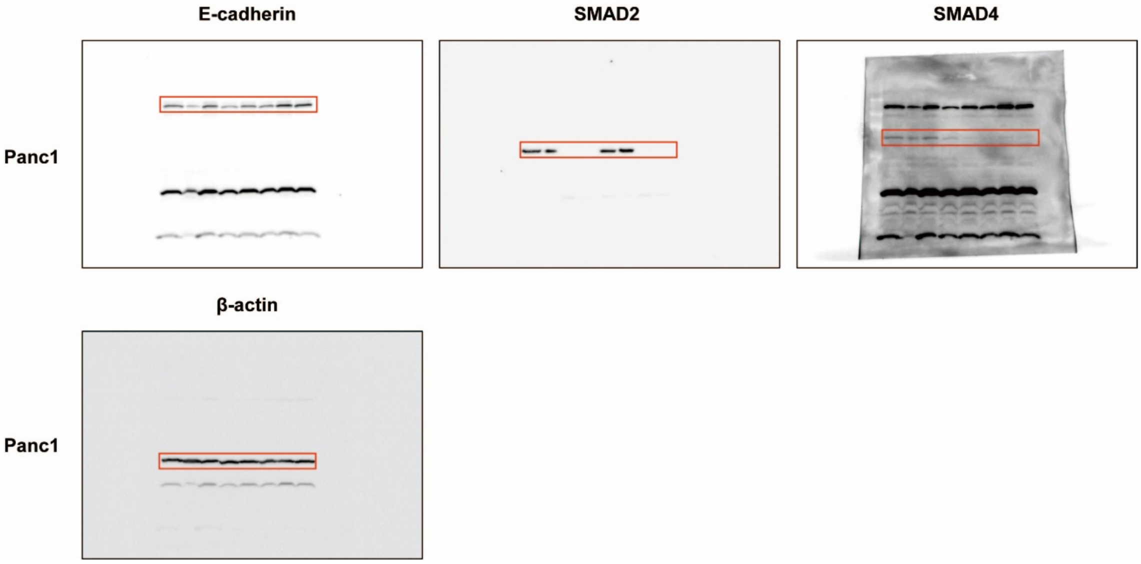

Supplement: Supplementary file 1 — Supplementary Information [file 41598_2017_4191_MOESM1_ESM.pdf]
